# Supplementary material for: Unraveling the impact of therapeutic drug monitoring via machine learning for patients with sepsis
Source: Cell Rep Med. 2024 Aug 9;5(8):101681. doi: 10.1016/j.xcrm.2024.101681 (PMC11384951; doi:10.1016/j.xcrm.2024.101681)
Supplement: Document S1. Figures S1–S3 and Table S1 [file mmc1.pdf]

Cell Reports Medicine, Volume *5*

Supplemental information

Unraveling the impact of therapeutic drug monitoring via machine learning for patients with sepsis

H. Ceren Ates, Abdallah Alshanawani, Stefan Hagel, Menino O. Cotta, Jason A. Roberts, Can Dincer, and Cihan Ates

rno	Visite_day	heathrate_min	heathrate_max	blood_pressure_min	blood_pressure_max	mean_art.bloodpressure_min	mean_art.bloodpressure_max	temp_min	...	Height	Weight
Ta-01-0001-3	['2017-01-26', '2017-01-27', '2017-01-28', '20...	['[46.0, 59.0]', '[56.0]', '[56.0]', '[46.0]',...	['[116.0, 113.0]', '[110.0]', '[86.0]', '[94.0...	['[63.0, nan]', nan, nan, nan, nan, nan, nan, ...	['[121.0, nan]', nan, nan, nan, nan, nan, nan,...	['[46.0, 64.0]', '[64.0]', '[69.0]', '[59.0]',...	['[86.0, 83.0]', '[81.0]', '[86.0]', '[81.0]',...	['[32.2, 34.6]', '[35.4]', '[35.7]', '[36.5]',...	...	170.0	60.0
Ta-01-0002-1	['2017-01-27', '2017-01-28', '2017-01-29', '20...	['[76.0, 80.0]', '[83.0]', '[72.0]', '[71.0]',...	['[115.0, 96.0]', '[129.0]', '[105.0]', '[95.0...	['[81.0, nan]', nan, nan, nan, nan, nan, nan]	['[122.0, nan]', nan, nan, nan, nan, nan, nan]	['[56.0, 65.0]', '[59.0]', '[58.0]', '[58.0]',...	['[77.0, 79.0]', '[82.0]', '[87.0]', '[83.0]',...	['[34.3, 37.1]', '[36.8]', '[36.9]', '[37.2]',...	...	164.0	60.0
Ta-01-0003-9	['2017-03-22', '2017-03-23', '2017-03-24', '20...	['[88.0, 94.0]', '[79.0]', '[76.0]', '[67.0]',...	['[174.0, 129.0]', '[112.0]', '[98.0]', '[80.0...	['[86.0, nan]', nan, nan, nan, nan, nan, nan, ...	['[149.0, nan]', nan, nan, nan, nan, nan, nan,...	['[62.0, 62.0]', '[60.0]', '[73.0]', '[79.0]',...	['[91.0, 112.0]', '[92.0]', '[107.0]', '[111.0...	['[36.9, 37.0]', '[36.8]', '[37.1]', '[37.2]',...	...	170.0	140.0
Ta-01-0004-6	['2017-03-24', '2017-03-25', '2017-03-26', '20...	['[67.0, 76.0]', '[58.0]', '[65.0]', '[81.0, n...	['[142.0, 118.0]', '[109.0]', '[105.0]', '[133...	['[79.0, nan]', nan, nan, nan, nan, nan, nan, ...	['[192.0, nan]', nan, nan, nan, nan, nan, nan,...	['[61.0, 61.0]', '[53.0]', '[59.0]', '[67.0, n...	['[133.0, 97.0]', '[102.0]', '[96.0]', '[114.0...	['[36.4, 37.1]', '[36.2]', '[37.7]', '[37.1, n...	...	185.0	80.0
Ta-01-0005-4	['2017-04-20', '2017-04-21', '2017-04-22', '20...	['[88.0, 84.0]', '[62.0]', '[60.0]', '[60.0]',...	['[178.0, 110.0]', '[87.0]', '[73.0]', '[89.0]...	['[78.0, nan]', nan, nan, nan, nan, nan, nan, ...	['[129.0, nan]', nan, nan, nan, nan, nan, nan,...	['[50.0, 56.0]', '[56.0]', '[56.0]', '[58.0]',...	['[76.0, 72.0]', '[84.0]', '[83.0]', '[106.0]'...	['[37.7, 36.5]', '[36.5]', '[36.4]', '[36.8]',...	...	155.0	55.0

Figure S1. Example of a master data frame for patient records, related to Fig. 1b-c. Only the first 11 features of 5 patients are shown in the table. Herein, each row represents a patient, and each column contains variable length lists of features. The shape of the data frame at this step was (254,152), denoting 152 features for 254 patients. First 11 column of the data is depicted below to demonstrate the df structure.

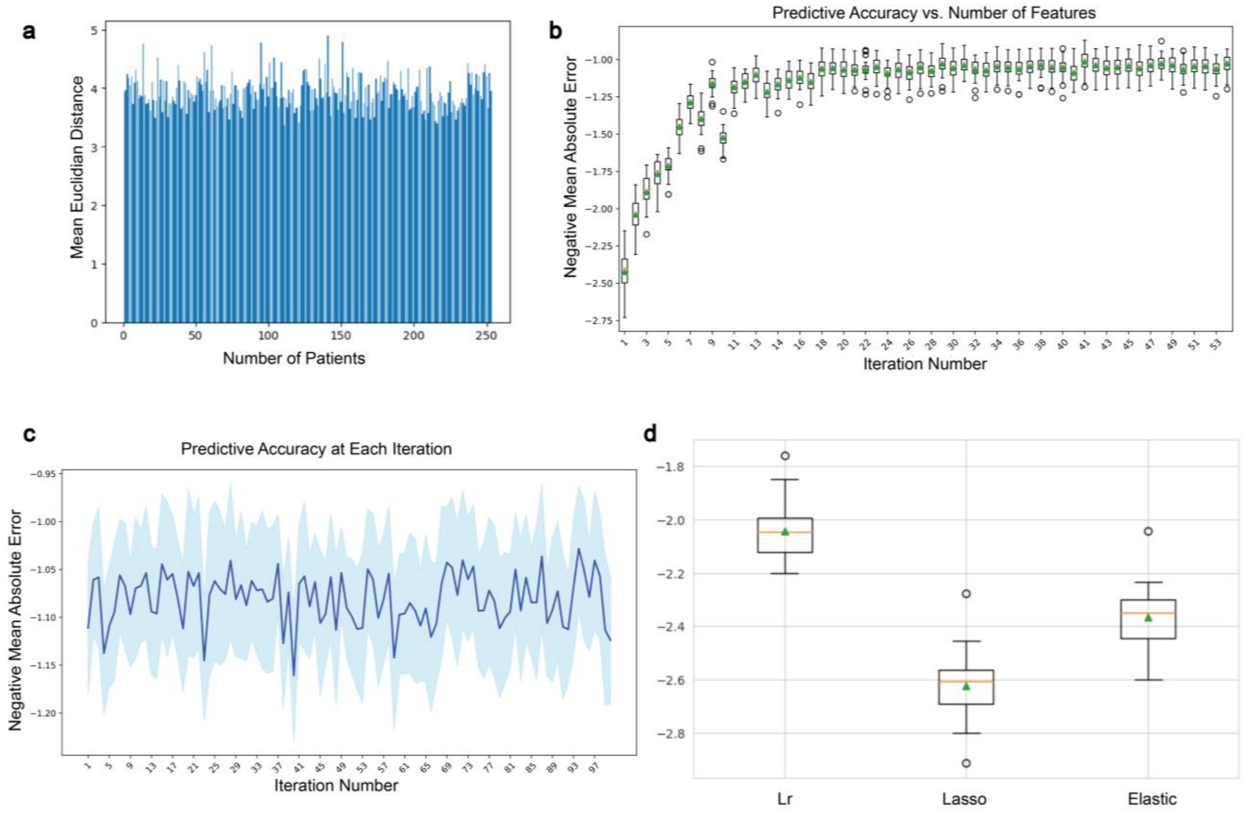

Figure S2. Details of similarity analysis and feature selection using decision model, related to Fig. 1-4 **a)** Mean pairwise distance in high dimensional feature space between patients for the day of admission. **b)** Predictive accuracy of the estimator with increasing number of features. For Scenario A, y axis denotes the -MAE of predicting the patient's SOFA score given the feature subset in repeated k-fold analysis. The predictive accuracy of the feature subset yielded such plateaus in all feature selection scenarios A-D, indicating that adding more features do not contribute to the model accuracy after a threshold, due to the limitations dictated by the sparsity of the dataspace. What differed between scenarios A-D was the negative MAEs, and the number of features it yields for its maximum accuracy. Overall, scenario A yielded the highest accuracy at lower number of features. **c)** Variations in repeated k-fold analysis for feature selection with GA (number_of_feature = 23). Each point shows the -MAE of the last generation of each 100 runs. This analysis further enables us to investigate the frequency of features being selected during the stochastic evolutionary process. **d)** Impact of using implicit feature selector as an estimator in GA wrapper. y axis denotes the -MAE in SOFA score predictions. lr: linear regression.

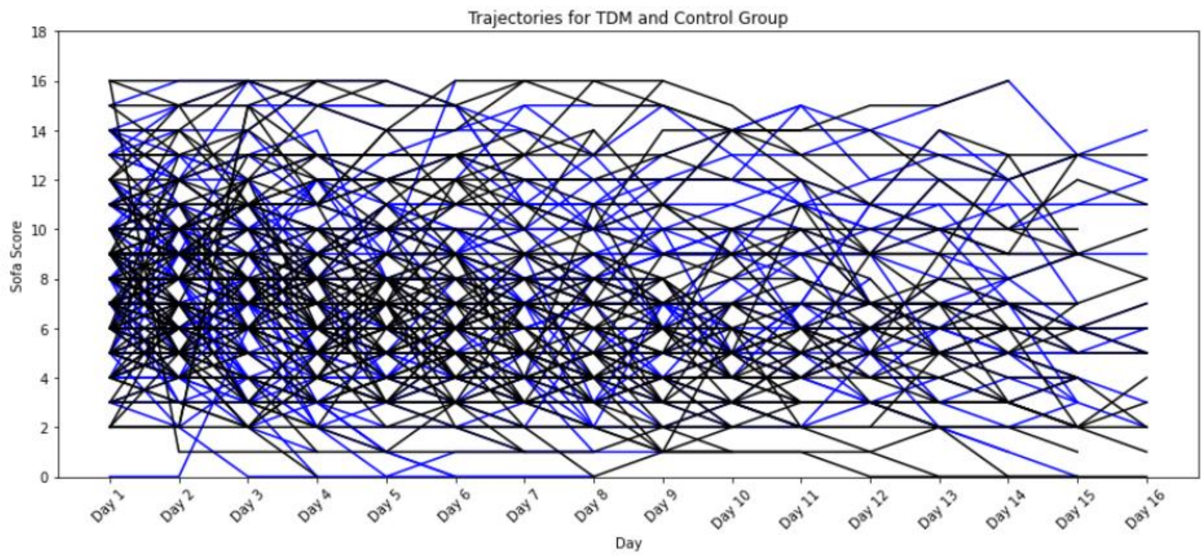

Figure S3. Fluctuations in patient SOFA score trajectories, related to Fig. 3. Patient health state jumps significantly between SOFA scores on a daily basis. Each curve represents a patient. Blue: TDM, Black: control.

Table S1. Detected pathogens data including type of pathogen and their occurrence in control and intervention groups responsible for sepsis episodes. Related to Fig. 4c-d. We fed the pathogen data to the decision model and found that pathogen-related data didn't make the cut very often, less than 10% of the time. But for the final feature set, we took the ones that were picked more than 10% of the time and threw them into another round of GA to make sure we covered any potential connections between them. Table is adapted from the original clinical study[2].

	**All patients[a] (n=253)**	**TDM (n=126)**	**No-TDM (n=127)**
**Gram-positive pathogen, No (%)**			
Staphylococcus aureus, Methicillin-susceptible	34 (6.7)	17 (6.2)	17 (7.4)
Staphylococcus aureus, Methicillin-resistant	1 (0.2)	1 (0.4)	0
Coagulase-negative staphylococci Methicillin-susceptible	26 (5.1)	14 (5.1)	12 (5.2)
Coagulase-negative staphylococci Methicillin-resistant	6 (1.2)	5 (1.8)	1 (0.4)
Streptococcus pneumoniae	3 (0.6)	1 (0.4)	2 (0.9)
Enterococcus faecalis	31 (6.1)	14 (5.1)	17 (7.4)
Enterococcus faecium	32 (6.3)	18 (6.5)	14 (6.1)
Other *Streptococcus* species	33 (6.6)	22 (8.0)	11 (4.8)
Other[b]	18 (3.6)	11 (4.0)	7 (3.0)
**Gram-negative pathogen, No (%)**			
Escherichia coli	86 (17.0)	50 (18.2)	36 (15.7)
Klebsiella species	59 (11.7)	30 (10.9)	29 (12.6)
Proteus species	25 (5.0)	14 (5.1)	11 (4.8)
Enterobacter species	27 (5.3)	14 (5.1)	13 (5.7)
Pseudomonas species	31 (6.1)	18 (6.5)	13 (5.7)
Serratia species	8 (1.6)	4 (1.5)	4 (1.7)
Citrobacter species	11 (2.2)	8 (2.9)	3 (1.3)
Acinetobacter species	2 (0.4)	1 (0.4)	1 (0.4)
Haemophilus species	3 (0.6)	1 (0.4)	2 (0.9)
Stenotrophomonas maltophilia	12 (2.4)	3 (1.1)	9 (3.9)
Other[c]	26 (5.1)	16 (5.8)	10 (4.3)
**Fungi, No (%)**			
Candida albicans	13 (2.6)	7 (2.5)	6 (2.6)
Other *Candida* species	8 (1.6)	2 (0.7)	6 (2.6)
Aspergillus species	3 (0.6)	2 (0.7)	1 (0.4)
Other[d]	6 (1.2)	1 (0.4)	5 (2.2)
**Other[e], No (%)**	1 (0.4)	1 (0.4)	0

[a] Multiple responses per patient possible

[b] Other includes other gram-positive bacteria (e.g., *Bacillus* species, *Corynebacterium* species, *Listeria species, Nocardia species*).

[c] Other includes other gram-negative bacteria (e.g., *Legionella*, *Moraxella*, *Neisseria*, *Salmonella*)

[d] Other includes *Phycomyces* species, *Coccidioides*, *Zygomyces*, *Rhizopus*, *Mucor*, and *Microsporum* species.

[e] Other includes *Mycobacteria species, Chlamydia species, Mycoplasma species*
