# Supplementary material for: Unraveling the impact of therapeutic drug monitoring via machine learning for patients with sepsis
Source: Cell Rep Med. 2024 Aug 9;5(8):101681. doi: 10.1016/j.xcrm.2024.101681 (PMC11384951; doi:10.1016/j.xcrm.2024.101681)
Supplement: Document S2. Article plus supplemental information [file mmc2.pdf]

Cell Reports
Medicine

Article

Unraveling the impact of therapeutic drug monitoring via machine learning for patients with sepsis

Graphical abstract

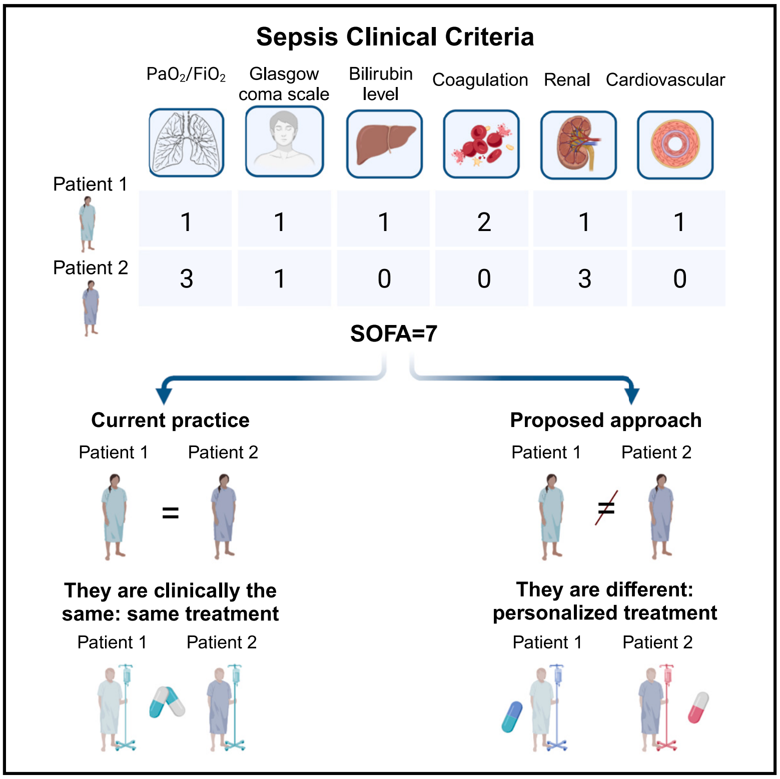

Highlights

- The impact of TDM on patient recovery is measured using machine learning
- The proposed data-driven framework enables dynamic tracking of patient drug response
- The study uses data from a clinical trial involving 248 patients with sepsis
- TDM-guided piperacillin/tazobactam therapy is proven to improve recovery rates

Authors

H. Ceren Ates, Abdallah Alshanawani, Stefan Hagel, Menino O. Cotta, Jason A. Roberts, Can Dincer, Cihan Ates

Correspondence

dincer@imtek.de (C.D.),
cihan.ates@kit.edu (C.A.)

In brief

Ates et al. propose a machine learning approach to measure the impact of therapeutic drug monitoring (TDM) on sepsis recovery. Their framework dynamically tracks treatment efficacy and patient response, using clinical trial data comparing TDM-guided piperacillin/tazobactam therapy to fixed dosing, demonstrating TDM's positive impact on patient recovery.

Ates et al., 2024, Cell Reports Medicine 5, 101681
August 20, 2024 © 2024 The Authors. Published by Elsevier Inc.
https://doi.org/10.1016/j.xcrm.2024.101681

CellPress

Cell Reports Medicine

CellPress OPEN ACCESS

Article

Unraveling the impact of therapeutic drug monitoring via machine learning for patients with sepsis

H. Ceren Ates,[1,2] Abdallah Alshanawani,[2] Stefan Hagel,[3] Menino O. Cotta,[4] Jason A. Roberts,[4,5,6] Can Dincer,[1,2,9,*] and Cihan Ates[7,8,*]
[1]University of Freiburg, FIT Freiburg Centre for Interactive Materials and Bioinspired Technology, 79110 Freiburg, Germany
[2]University of Freiburg, Department of Microsystems Engineering (IMTEK), 79110 Freiburg, Germany
[3]Institute for Infectious Diseases and Infection Control, Jena University Hospital – Friedrich Schiller University Jena, 07747 Jena, Germany
[4]Faculty of Medicine, University of Queensland Centre for Clinical Research, The University of Queensland, Brisbane, QLD 4006, Australia
[5]Departments of Intensive Care Medicine and Pharmacy, Royal Brisbane and Women's Hospital, Brisbane, QLD 4006, Australia
[6]Division of Anaesthesiology Critical Care Emergency and Pain Medicine, Nîmes University Hospital, University of Montpellier, 34295 Nîmes, France
[7]Karlsruhe Institute of Technology (KIT), Machine Intelligence in Energy Systems, 76131 Karlsruhe, Germany
[8]Karlsruhe Institute of Technology (KIT), Center of Health Technologies, 76131 Karlsruhe, Germany
[9]Lead contact
*Correspondence: dincer@imtek.de (C.D.), cihan.ates@kit.edu (C.A.)
https://doi.org/10.1016/j.xcrm.2024.101681

SUMMARY

Clinical studies investigating the benefits of beta-lactam therapeutic drug monitoring (TDM) among critically ill patients are hindered by small patient groups, variability between studies, patient heterogeneity, and inadequate use of TDM. Accordingly, definitive conclusions regarding the efficacy of TDM remain elusive. To address these challenges, we propose an innovative approach that leverages data-driven methods to unveil the concealed connections between therapy effectiveness and patient data, collected through a randomized controlled trial (DRKS00011159; 10th October 2016). Our findings reveal that machine learning algorithms can successfully identify informative features that distinguish between healthy and sick states. These hold promise as potential markers for disease classification and severity stratification, as well as offering a continuous and data-driven "multidimensional" Sequential Organ Failure Assessment (SOFA) score. The positive impact of TDM on patient recovery rates is demonstrated by unraveling the intricate connections between therapy effectiveness and clinically relevant data via machine learning.

INTRODUCTION

Sepsis is a life-threatening condition that poses significant challenges to healthcare professionals due to its difficulty in early detection and management, leading to a high mortality rate. Intravenous antibiotic therapy, including the commonly used beta-lactam class of antibiotics, is a crucial element in the management of sepsis.[1] Antibiotic administration should start as soon as possible, ideally within the first hour of diagnosis and after clinical cultures are obtained.[2] Early recognition and optimized treatment of sepsis can improve the chances of patient survival. Due to the heterogeneous presentation of sepsis, however, early recognition is often challenging. This can lead to delayed care, increasing the risk of organ failure and negatively impacting patient outcomes.

Due to acute disease processes and treatment interventions associated with sepsis and its management in the intensive care unit (ICU), critically ill patients often experience altered pharmacokinetics (PK).[3–5] This can result in highly variable and unpredictable exposures of beta-lactam antibiotics.[6] Moreover, due to antibiotic usage being higher in the ICU compared to other areas of the hospital and in the community, pathogens isolated in ICU patients are at risk of reduced antibiotic susceptibility. This adds to the difficulty in ensuring beta-lactam antibiotic exposures attain desired pharmacodynamic (PD) targets.[7] Therapeutic drug monitoring (TDM) offers a potential solution to ensure antibiotic concentrations are maintained at target exposures throughout the treatment period. This intervention may help improve treatment failure rates and reduce the risk of exposure-related drug toxicity.

However, the wider adoption of beta-lactam TDM in ICUs is impeded by several challenges, including limited availability, operational complexities that can delay turnaround times for reporting results, as well as cost considerations.[8] As a result, healthcare professionals are compelled to carefully assess the optimal allocation of resources and prioritize patient groups that are likely to derive the greatest benefits from beta-lactam TDM.[9]

© 2024 The Authors. Published by Elsevier Inc.
This is an open access article under the CC BY license (http://creativecommons.org/licenses/by/4.0/).

Despite several randomized controlled trials investigating the impact of beta-lactam antibiotic TDM in the ICU,[2,10–13] none have yet demonstrated a significant difference in patient outcomes. To address this gap, it is important to approach the problem from a broader perspective, considering its multidimensional nature. This involves examining a wider range of outcomes including clinical cure, microbiological eradication, development of antibiotic resistance, patient morbidity and mortality, as well as conducting rigorous cost-effectiveness analyses. By considering and analyzing such multifaceted information, a deeper understanding of how to optimize beta-lactam antibiotic dosing strategies in critically ill patients can be obtained.[5,7,14]

However, understanding and processing such large-dimensional and heterogeneous data is not straightforward with conventional methods. In this context, machine learning (ML) emerges as a powerful tool to navigate these complexities. By harnessing ML, informative features can be identified from the collected multidimensional, temporal patient data, enabling the creation of a comprehensive patient state representation (Figure 1A). This learned representation facilitates the distinction between healthier and relatively sicker states by comparing informative measured features over time within a patient and across different patients. Such data-driven analyses offer the means to monitor patients' recovery trajectories and treatment responses, shedding light on the intricate interplay between therapy, patient dynamics, and outcomes. The inclusion of ML-based methodologies thus provides a crucial lens through which to quantify the impact of TDM on patient recovery, enhancing our ability to derive meaningful insights from the intricate web of clinical data. The objective of this work is to quantitatively analyze the influence of TDM on recovery trajectories, with specific attention to three key aspects: (1) quantification of the patient state during piperacillin/tazobactam antibiotic therapy, (2) the impact of piperacillin/tazobactam antibiotic TDM on patient state dynamics, and (3) the effect of piperacillin/tazobactam TDM on the survival of patients (Figures 1B and 1C).

RESULTS

Data-driven assessment of TDM and control group split

We first computed the mean pairwise Euclidian distances in the original feature space, which yielded no difference between the TDM and control group patients (Figure S2). Considering the small number of patients in the study (n = 248), we further extended the similarity analysis in lower dimensional representation generated by two dimensionality reduction techniques, t-distributed stochastic neighbor embedding and linear principal component analysis (Figures 2A and 2B). In both cases, computed pairwise distance statistics and their distribution were the same. The analysis of the pathogen reports for the first day was also aligned with this data-driven deduction. Figures 3D and 3E demonstrate the presence of distinct pathogen types in both the TDM and control group on day 1 (following randomization). Notably, the pathogen distributions exhibited similarity between the two groups. Furthermore, the distribution of piperacillin-resistant (Figure 3D) and sepsis-causing pathogens (Figure 3E) followed the same pattern. Therefore, it was deduced that patients in both the TDM and control sub-populations started treatment in similar conditions, enabling the conduction of the proposed state-space trajectory analysis objectively.

Impact of TDM on patient state trajectory

The evolution of the patient states for the TDM and control group populations revealed three important outcomes (Figure 2C). At the beginning of the study, both the TDM and control groups demonstrated a comparable distance to the reference state space (i.e., patients with Sequential Organ Failure Assessment [SOFA] score = 1), indicating similar feature distributions within the patient groups. This finding aligned with the results illustrated in Figures 2A and 2B, emphasizing the resemblance in patient statuses between the control and TDM groups during the initial phase of the study. Secondly, as treatment continued, the TDM groups "moved" faster toward the reference state compared to the control group. In particular, the distance between the TDM and control groups was found to be the greatest between 48 and 72 h after the randomization. It should be noted that all patients received the same dosage at the beginning of the clinical study (day 1), and dose adjustments were made once the data were available for the TDM group on day 2. The movement after dose adjustment (day 3 and 4) toward the reference state quantitatively demonstrated that the effective distance traveled per day was much higher for the TDM group. Thirdly, similar trends were observed between days 7 and 10, indicating a stable recovery rate toward a healthy state (SOFA 1) for both groups. Considering the importance of the treatment in the first 72 h of sepsis on one hand, the difference in the slope of the curves for the number of people who left the study deceased up to day 10 (Figure 3B). On the other hand, we may argue two modes of response to treatment in the patient groups. During the first three days, precise dose adjustments were crucial for sepsis management, where TDM leads to improvements in two modes. Some patients experienced immediate short-term benefits, leading to a higher proportion of TDM patients exiting the ICU alive within the first week (Figure 3A). Conversely, standard treatment appeared to prolong recovery time for those who survived (Figure 3A). A second mode of TDM impact was evident in the overall treatment trajectory, particularly visible in Figure 3B. Herein, the control group exhibited a relatively steeper slope. This observation can be explained as a long-term benefit of TDM, enhancing recovery prospects for patients requiring extended treatment periods, which in turn results in lower mortality.

The statistical analysis of the data presented in Figures 3A and 3B was done by comparing the cumulative distribution function (CDF) and probability density function (PDF) curves for TDM and control patients to assess the impact of time on the recovery process. Accordingly, we computed the Kolmogorov-Smirnov test statistic for the CDF, which explains whether two samples (TDM and control) came from the same distribution or not. They were found as 0.214 and 0.286 for Figures 3A and 3B, respectively. We also computed the Hellinger distances between the PDFs, yielding 0.245 and 0.432 for Figures 3A and 3B, respectively. Results indicate a relatively large degree of dissimilarity particularly for Figure 3B, supporting the argument that TDM appears to impact the overall recovery rates of patients.

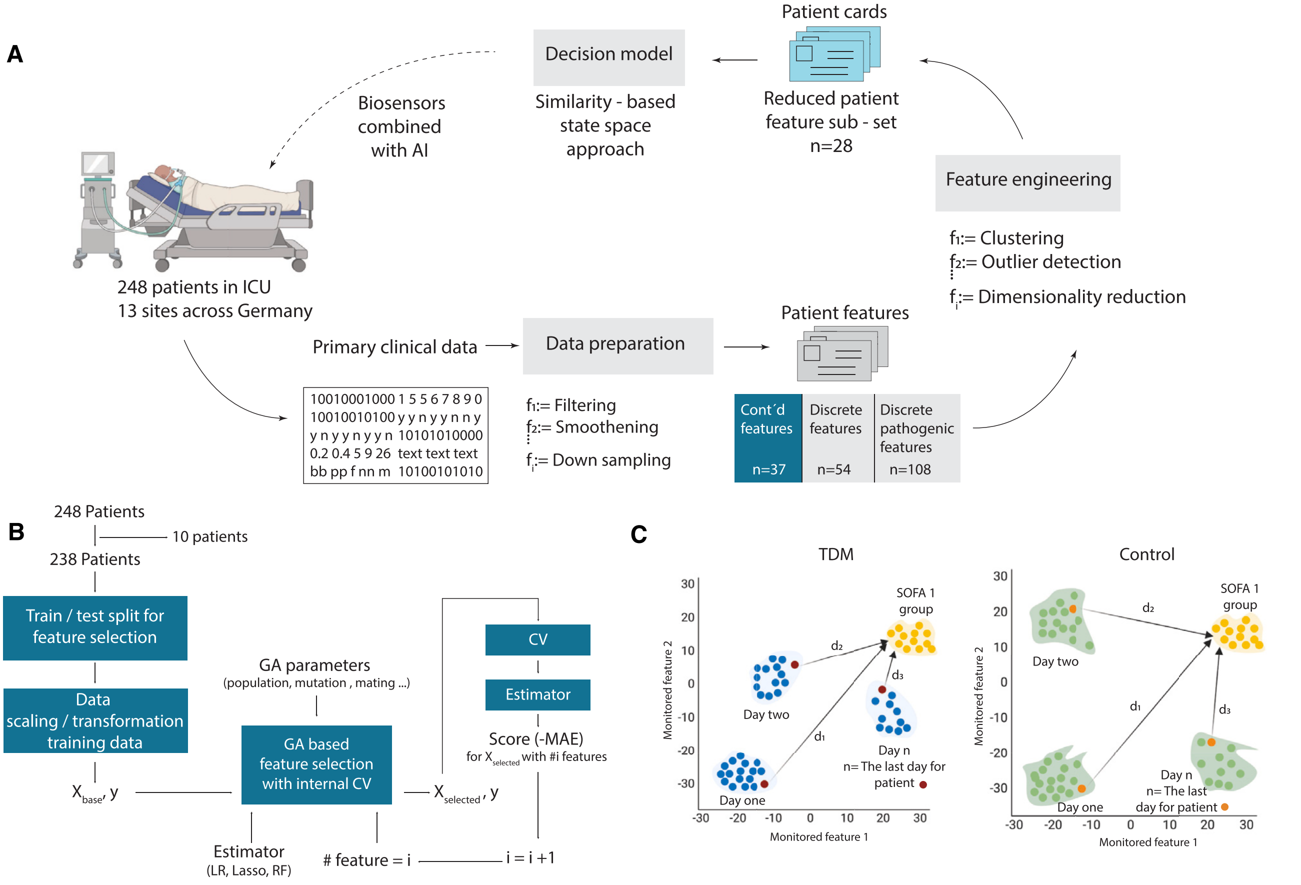

Figure 1. Quantifying effect of TDM by a similarity-based state-space approach

(A) Data processing and feature selection pipeline for patient status analysis using the in-house data preparation code. The heterogeneous medical database collected from hospitals is transformed into digital patient cards, followed by the selection of the top 28 representative features through feature engineering. A similarity-based state tracking approach is employed to compare the impact of TDM on patient status. The integration of biosensors for frequent sampling and enhanced drug dosage control is proposed to complete the loop and further optimize patient care.

(B) Feature selection workflow utilizing genetic algorithm (GA) implementation. The process involves leaving out 10 patients for generalizability testing, train/test split for feature selection evaluation, feature scaling/transformation, and iterative refinement with GA with cross-validation. The final feature set is determined through a frequency analysis of 100 repetitions.

(C) Visualization of traveled distance analogy in a 2D feature space to assess patient state dissimilarity. The blue and orange points represent TDM and control group patients, respectively. The distance to the reference health state (d_i) indicates the degree of dissimilarity from the "healthy" state. Patients in both groups start their recovery trajectory in a specific sub-space of the 2D state space and are expected to move toward the reference state over time. The rate of recovery is determined by how quickly the groups progress from their initial states to the reference "healthy town" of Sequential Organ Failure Assessment (SOFA) score of 1. The cumulative sum of Mahalanobis distances is calculated to quantify the difference between TDM and control groups' proximity to the healthy zone for each day.

When the status of the patients leaving the study alive was further examined, it is seen that the last recorded SOFA scores were also lower for the TDM group for the first 10 days, with the exception of day 1 in which 2 patients left the study with a high SOFA score (Figure 3C). Statistical analysis of the last day SOFA scores for patients who left the study alive (day <11) was done via ANOVA test. p value here was found as 0.0314, indicating that the impact of TDM on the last day SOFA scores is visible. This is also consistent with the fact that in the original statistical analysis of the study, a lower mortality rate and a higher clinical and microbiological cure rate were observed in patients receiving TDM-guided therapy.

The statistical test for last day SOFA scores was done for data points up to day 11 due to the decrease in the number of patients remaining in the study. We postulate that the observed "spike" in SOFA scores between day 12 and 14 among patients who left the clinical study alive within the TDM group can be attributed to two related factors. Firstly, the remaining smaller population size in TDM group could potentially magnify the impact of individual variations in patient responses. Secondly, a relatively higher proportion of critically ill individuals remained in the TDM group compared to the control as a result of the TDM group's overall lower mortality rate throughout the first 10 days.

Randomly sampled individual patient trajectories from the TDM and control group (Figure 2D) further revealed unique responses to the therapy, highlighting the individualistic nature of the treatment process and the need for an individualized therapy management for a better recovery. It is noteworthy that the dose

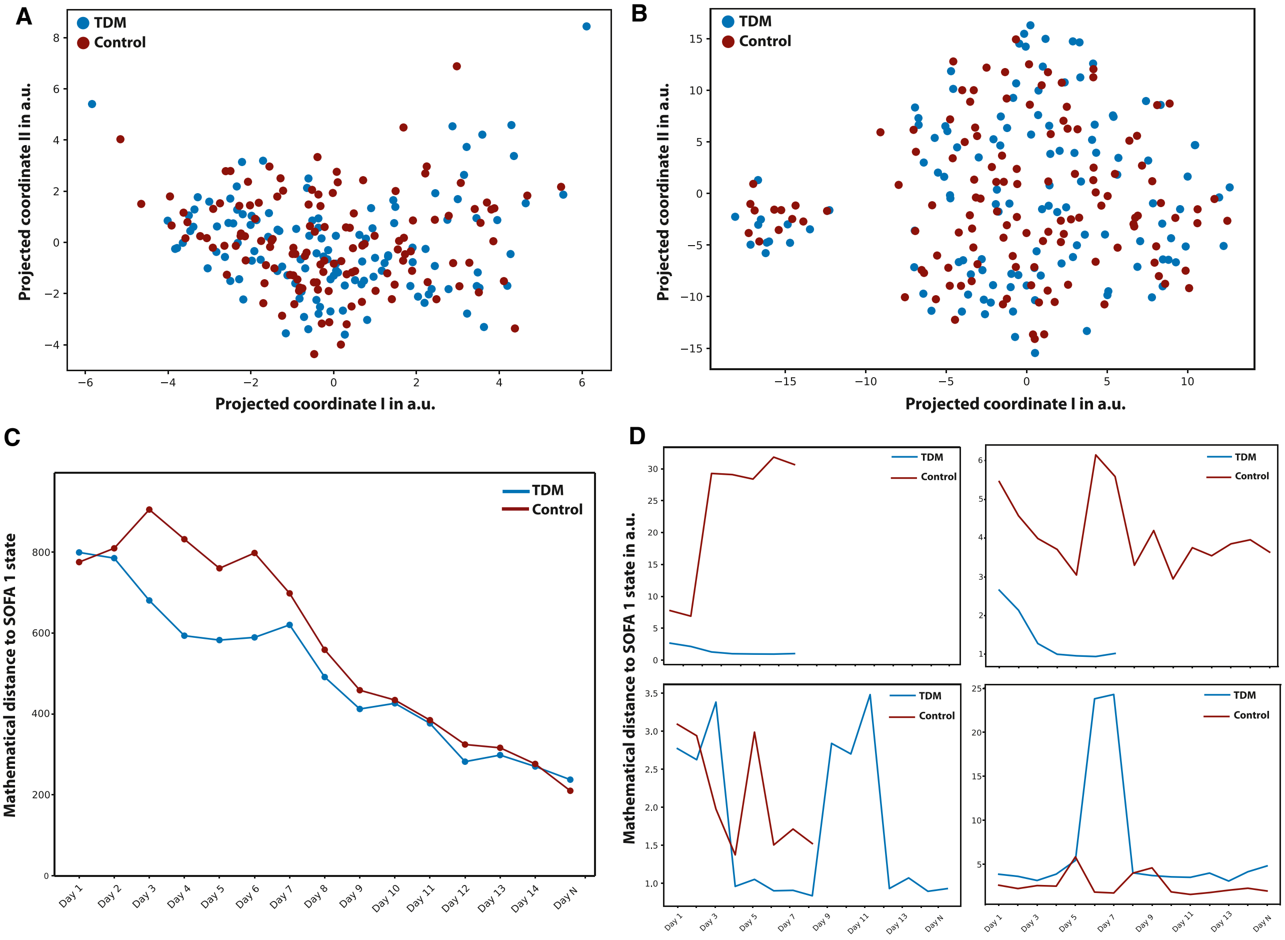

Figure 2. Data-driven assessment of TDM and the impact of TDM on patient state trajectory

The underlying hypothesis of this study is that the medical data collected daily during the clinical study hold valuable information regarding the patients' health states. By employing mathematical techniques of similarity, the gradual changes in patient states based on the distribution of their health states were quantified.

(A and B) Mathematical similarity of patient groups on day 1 is demonstrated using two dimensionality reduction techniques: (A) t-distributed stochastic neighbor embedding (t-SNE) and (B) principal component analysis (PCA). Both t-SNE and PCA embeddings indicate that patient states were homogenously distributed on the day of admission, validating the random TDM and control split. Quantitative comparison in high-dimensional feature space is given in the supplementary information.

(C) The concept of "traveling to a healthier state," which is evaluated by calculating the normalized Mahalanobis distance between the patient's health status on each day and the reference state.

(D) Randomly sampled individual patient trajectories from both the control and TDM groups, revealing the disparity in mathematical distance toward the SOFA 1 state.

adjustment frequency in the TDM group remained suboptimal for the individuals, limited to 24-h cycles of drug concentration data. These findings underscore the need for further investigation with more frequent dose adjustments to enhance treatment efficacy and bolster confidence in the observed outcomes.

DISCUSSION

TDM is the practice of measuring, analyzing, and adjusting the drug levels in a patient's blood to achieve the desired therapeutic outcomes while avoiding adverse effects. Here, the drug concentrations gauged at specific intervals provide valuable information about a patient's individual PK/PD, allowing the healthcare providers to tune treatment regimens based on the individual state and make "informed" decisions regarding dosage adjustments or changes in medication. Therefore, the success of TDM practices strongly depends on how frequently and accurately the drug concentrations are measured and how the patient current state is correlated with the therapeutic window. Our study utilizes data collected in a clinical trial conducted across 13 sites, where half of the patients were subjected to dose adjustment. Notably, our findings demonstrate the capability of ML algorithms to discern meaningful features, allowing us to quantitatively capture the patient recovery process and gain valuable insights into therapy progression.

Despite the rich clinical data regarding patient state (199 features), however, the dynamic TDM process was only run using a daily granularity. That is, the dose adjustment in the beta-lactam

CellPress
OPEN ACCESS

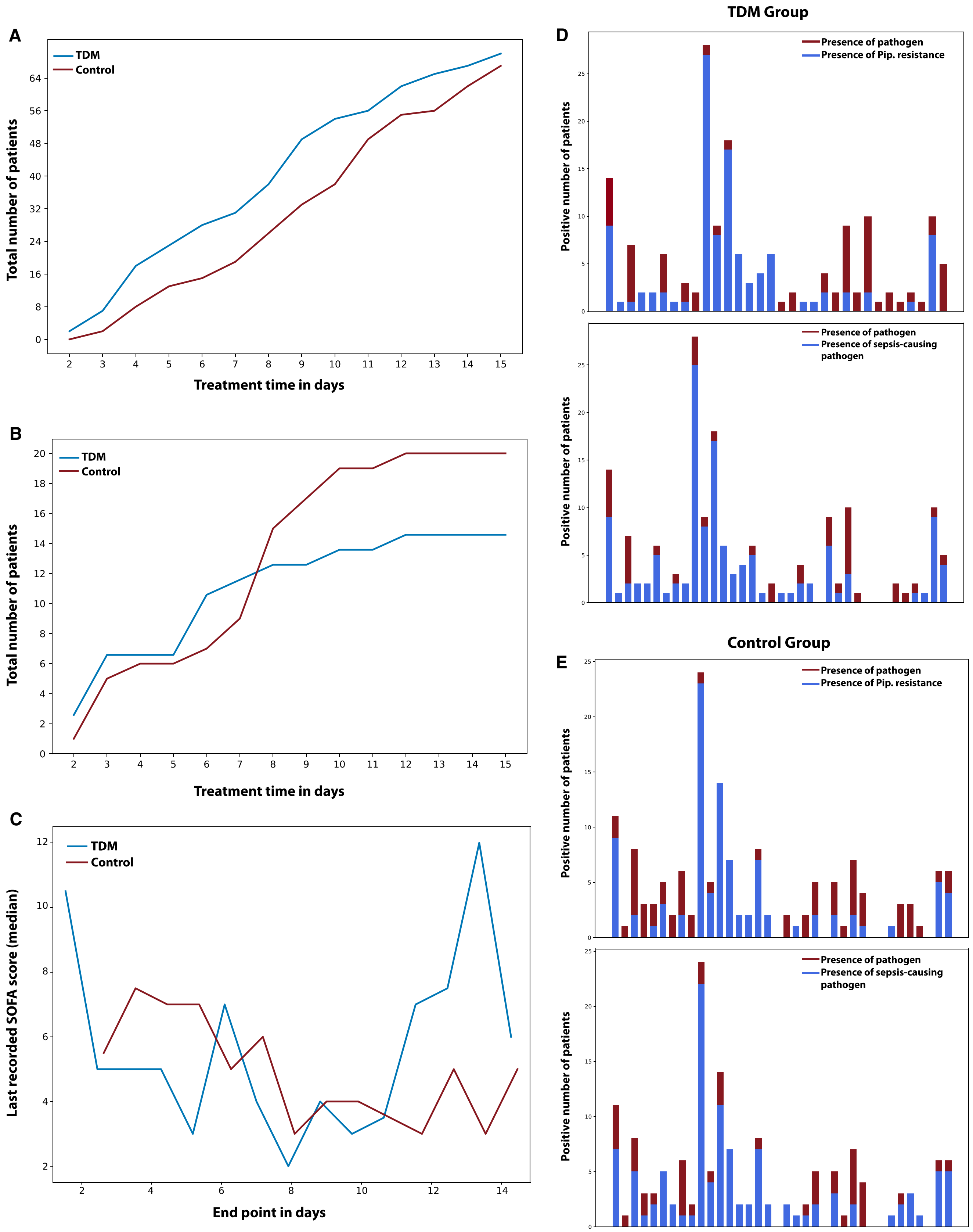

(legend on next page)

TDM group was made once a day; thus, smaller time interval fluctuations in drug concentrations could not be captured. Likewise, of the piperacillin concentrations measured in the TDM group, 88.1% (n = 510) were reported on the same day, while 10.0% (n = 58) were reported at a later date and 1.9% (n = 11) were never reported.[2] As a result of this "process delay," TDM feedback cycle used to adjust the dosage regimen was potentially slower when compared to the evolution of the patient state, which can be seen in the daily SOFA score fluctuations (Figure S3). Another limitation of the clinical study was the limited number of dosage adjustments in the TDM group. Hence, the dose adjustment may not have been made at the optimum time for the patients. For instance, at day 1, for only 70% of the TDM patients, dose adjustment was considered necessary, followed by an average of 48% during treatment, and ended with less than 30% for day 9 and day 10. Four patients in the TDM group had never received a dose adjustment.[2]

The other limitation of the TDM workflow applied was its dependency on the minimum inhibitory concentration (MIC),[2] which represents the lowest concentration of a drug that is required to inhibit the growth of the microorganism in a laboratory setting. In the TDM patients, the dose adjustment was based on the most recent pathology report and the corresponding MIC values. Nonetheless, setting the therapeutic window based on MIC has two important limitations: (1) serum drug concentrations do not necessarily represent the concentration at the site of infection; hence, drug concentrations above the MIC may not have been reached at the site,[15] and (2) complex PK/PD of the patient status may demand a drug concentration different than the MIC. As the MIC value only distinguishes between the growth and suppression of the pathogen under lab conditions, the drug concentration recommended by the MIC may not be sufficient to kill the pathogen at the site of infection. In an ideal scheme, MIC values can be used to set the initial therapeutic window, which is continuously updated based on an individualized patient model stemming from the site of infection.[8,16] However, despite these challenges, ML-augmented analysis of the patient data conclusively demonstrated that implementing dose adjustment policies had a significant and favorable impact on the overall recovery of patients in the TDM group. In particular, dose adjustment based on TDM had the largest impact within the first 72 h of admission, which is noted as an important treatment window in the management of patients with sepsis.

In TDM, quantifying the impact of defined drug concentrations on a patient's recovery trajectory is vital. In the clinical case being examined, for instance, updating patient data on an hourly basis, rather than daily, could significantly amplify the impact of TDM, especially within the first 72 h. By doing so, it would have been possible to determine the most effective drug concentration to target, ensuring the best possible outcomes for the patient. However, this necessitates (1) the identification of the optimal free drug concentration and (2) a quantitative description of the patient's state. In this regard, the use of biosensors can potentially provide accurate, on-site, and rapid detection of the drug concentration from both blood and non-invasive bodily fluids including sweat, saliva, tear, and breath, providing a chance to accelerate the response time of the TDM cycle.[17–22] While still in the research stage, these devices are engineered to offer frequent measurements, detect subtle changes in drug concentration, and deliver results that are both sensitive and consistent. A key advantage of biosensors lies in their ability to operate with minimal volumes of various biofluids, such as blood obtained through a finger prick or non-invasive samples. This capability facilitates frequent sampling and measurement, thereby enhancing the precision of drug monitoring.[15] Furthermore, the multiplexing functionality of biosensors enables the simultaneous monitoring of multiple analytes and/or samples, improving diagnosis capabilities and establishing a better individual reference state.[22,23] Additionally, this sensor data can be utilized to build digital patient models, which can further enable model predictive control policies via simulating the patient response with individualized PK/PD parameters. By integrating population-specific antibiotic PK models with patient-specific information such as kidney function, weight, pathogen data, and TDM results, tailored dosing regimens can be calculated via model-informed precision dosing software.[8,16,18] Preliminary findings indicate that such a personalized approach improves the attainment of PK/PD targets, particularly for patients at high risk of mortality from infections.[24] When coupled with data-driven methods, biosensors have the potential to improve therapy efficacy by providing a more accurate representation of the therapeutic index, tailored to individual patients' needs. This personalized approach enables the customization of dosage regimens, optimizing treatment outcomes.

Recent developments in the wearable body area network,[25] where multiple wearables mounted on different parts of the body to concurrently analyze various physiological markers, and the integration of internet of things devices into healthcare monitoring[26] are enabling continuous feature collection possible for quantitative description of the patient's state. By leveraging ML capabilities, voluminous sensor data, together with the previous medical records, genetic information, and individualized healthy reference state, data-driven algorithms can continuously adapt and refine dose adjustment strategies and the pharmacokinetic models in real time.

Figure 3. Effect of TDM on patient recovery trajectories

(A) The number of people left the study alive in both control and TDM groups.

(B) The number of people left the study dead in both control and TDM groups.

(C) Last recorded SOFA scores (median) for patients left the study alive in both the TDM and control groups.

(D) The presence of distinct pathogen types (red) with the distribution of piperacillin-resistant pathogen (blue) in both the TDM and control groups on day 1 (following randomization).

(E) The presence of distinct pathogen types (red) with the distribution of sepsis-causing pathogen (blue) in both the TDM and control groups on day 1 (following randomization). Pathogen distributions and the distribution of piperacillin-resistant and sepsis-causing pathogens exhibit similarities between the two groups, indicating that the patients in TDM and control sub-populations started the treatment in similar conditions (see Table S1).

CellPress
OPEN ACCESS

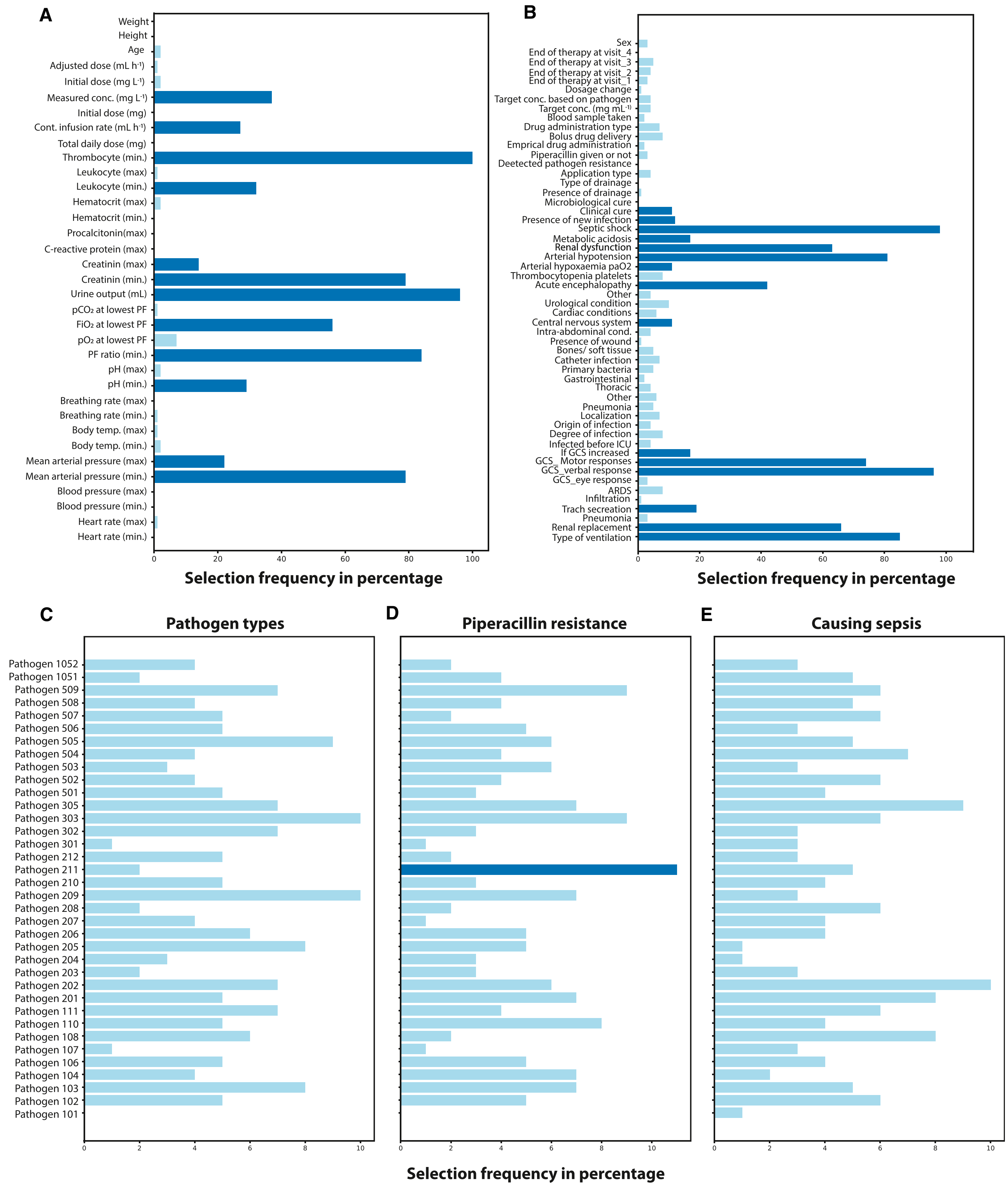

Figure 4. Features considered relevant by the evolutionary feature selection algorithm

Each bar denotes how many times a feature was picked by GA for SOFA score prediction. Features used in the patient state analysis are highlighted as dark blue.

(A) Continuous features encompass various information (such as age, height, and body weight), laboratory (leukocyte count, hematocrit levels, and creatinine levels), drug-related information (concentration and infusion rate), and physiological measurements (such as breathing rate, body temperature, and mean arterial pressure).

(legend continued on next page)

Another key role that wearable technology can play is the definition of an individualized reference healthy state. Our analysis showed that patient states can be defined from measured physiological parameters and observations, and the effect of therapy can be quantified by comparing the instantaneous patient state with a reference "healthy state." In the current implementation, we used the SOFA = 1 patient states as reference distribution, and closeness to this reference distribution provided a proxy measure for "being healthy" for all patients. In a 4P (predictive, preventive, personalized, and participatory) medicine concept enabled by wearable sensors,[25,27] individualized healthy states for patients can be learned, which would further increase the accuracy of state space tracking of health status. Such advancements underscore the importance of collaborative efforts among clinicians, technology developers, and data scientists. By emphasizing the significance of interdisciplinary collaboration, this paper aims to underscore the importance of leveraging advanced technologies to enhance patient care and outcomes in TDM.

The proposed analysis in this study also reveals an intriguing finding regarding the clinical relevance of the selected feature set and its relationship to the SOFA score. The features commonly used to calculate the SOFA score in clinical practice, such as thrombocytes, urine, creatinine, the Glasgow Coma Scale (GCS), and encephalopathy, were also found to be informative in this study. From a data science perspective, the calculation of the SOFA score can be viewed as a rule-based technique that reduces the dimensionality of the data, transforming a 28-dimensional vector into a single scalar value. This interpretation leads to two practical outcomes. First, it highlights the need to understand the limitations of the SOFA score analysis and how it can be interpreted. Second, it sheds light on the proposed state-space patient trajectory analysis, which can be seen as a high-dimensional, continuous version of the SOFA score.

At the first glance, reduction of all clinically relevant data as the SOFA score can be considered as a practical way for interpretation. However, the way SOFA scores are calculated in current practice can result in inaccurate clustering or classification of patients with different status. The SOFA score assesses the functionality or degree of failure in six key systems: respiratory, cardiovascular, hepatic, coagulation, renal, and neurological. It is important to recognize that while two patients may have the same SOFA score, their clinical situations differ significantly. For instance, one patient may have impaired renal function resulting in their score of 4, whereas another patient may experience minor issues across four different major systems. Therefore, interpreting SOFA scores requires careful consideration of the specific organ systems involved to obtain a comprehensive understanding of each patient's condition.

To address these limitations, our analysis provides a way to maintain a "multidimensional" SOFA score based on Euclidean distances. This allows for a quantitative assessment of the mathematical similarity between patient states while preserving the maximum amount of information. Hence, the method proposed in this study can be interpreted as a continuous and extended form of the SOFA score analysis. This proposed approach can be considered as a "live map," showing the state of the patient, with respect to a reference healthy state distribution. By utilizing this approach, it becomes possible to distinguish between patients with the same SOFA score but different physiological states. This enhanced level of differentiation provides valuable insights for clinical decision-making and patient management. With such a tool, the added value of adaptive policies such as TDM compared to pre-determined dosing strategies can also be measured with ease, as it enables a quantitative feedback system. For a potential TDM application, however, at least one of the following should be true: (1) there must be a narrow margin for an effective dosage, such that there is a risk of underdose (i.e., risk of antibiotic resistance)/overdose (risk of toxicity) treatment, (2) clinically effective drug concentration or drug mix is changing significantly for individuals and cannot be known *a priori*, or (3) the dose regimen is to be dynamically changed during a treatment, and a patient response is needed to be tracked. In such cases, proposed methodology can be followed, as it gives a quantitative, objective measure of the treatment process.

The "multidimensional" SOFA score approach represents an alternative interpretation of existing clinical data, offering seamless integration into established frameworks. Leveraging similarity-based learning, a well-established domain within ML, our approach utilizes readily available functions and methods across various programming languages. Conceptually, our approach can be likened to a high-dimensional map, allowing for the tracking of a patient's progress across multiple dimensions. Specifically, it enables assessment with respect to (1) their reference state if it exists (e.g., previous routine checkup measurements), (2) their state compared to their previous measurements during the treatment, and (3) a clinical database for a relevant fraction of a population (gender, age, known diseases, etc.). Deploying the proposed methodology and the interpretation of the results facilitated through a user-friendly graphical interface are both straightforward, when reference states are accessible. Utilization of the proposed approach would be very similar to SOFA score practices, however, with the advantage of keeping all the relevant information about patients (multidimensional) in a continuous, distinctive framework (instead of using discrete, overlapping ordinal SOFA classes).

Our study has revealed significant findings with implications for ML-augmented disease classification, patient stratification, and monitoring treatment response. Features identified with ML techniques accurately reflected the recovery process of patients with sepsis in ICUs, providing valuable insights into therapy progression and effectiveness. Importantly, continuous monitoring of these features enabled precise measurement of the recovery rate, emphasizing their potential as indicators of

(B) Discrete features consist of yes/no questions and ordinal variables, such as the presence of metabolic acidosis, renal dysfunction, or the need for renal replacement.
(C) Whether a pathogen could be detected in the patient. Microbiology reports cover 36 different pathogens, including gram-positive and gram-negative bacteria, fungi, and other pathogens such as Chlamydia species; (D) whether a detected pathogen is resistant to piperacillin and (E) whether the pathogen type is responsible for the sepsis episode. See Table S1 for more details.

treatment response. By using artificial intelligence, we demonstrated quantitatively that beta-lactam antibiotic TDM implementation leads to higher recovery rates and enhanced patient outcomes. Our findings highlight that the state between healthy and sick individuals can be differentiated from the temporal data, which in turn can be used to quantify the recovery process as a reliable measurement of the recovery rate. Additionally, TDM-guided dosing was found to significantly alter the trajectory of recovery, underlining its potential for personalized medicine and enhanced patient care.

Limitations of the study

The proposed methodology measures the mathematical dissimilarity (i.e., Mahalanobis distance[28,30–32]) between the current state of an individual patient at a given time and a reference healthy state distribution (SOFA = 1 states available in the clinical study). This is because the personalized healthy state of each individual is not available. In other words, statistics of the relatively healthier patients in the clinical study are used to define a reference state, which may not perfectly represent being healthy for each individual, introducing a sampling bias for both the control and the TDM group. To minimize the impact of such bias, aggregated Mahalanobis distances for the TDM and control groups are compared, and the impact of TDM is reported cumulatively for each group. The second limitation is the sample size, which included 248 patients and 2,376 state vectors in total. The study should be extended to a larger population for better generalization of the outcomes. The original study from which the patient data were taken did not include full demographic analysis of the patients. Although this information does not directly impact the analysis, its absence could be seen as a limitation.

STAR★METHODS

Detailed methods are provided in the online version of this paper and include the following:

- KEY RESOURCES TABLE
- RESOURCE AVAILABILITY
  - Lead contact
  - Materials availability
  - Data and code availability
- EXPERIMENTAL MODEL AND STUDY PARTICIPANT DETAILS
  - Study design and objectives
  - Details of the clinical study
- METHOD DETAILS
  - Patient state and measure of similarity
  - Feature engineering and selection
  - SOFA score and patient analysis
- QUANTIFICATION AND STATISTICAL ANALYSIS
  - Kolmogorov-Smirnov (KS) test
  - Hellinger distance
  - Multiple factor analysis of variance
- ADDITIONAL RESOURCES
  - Data preparation procedure
  - Pairwise distance analysis on day 1
  - Details of feature selection via wrapper

SUPPLEMENTAL INFORMATION

Supplemental information can be found online at https://doi.org/10.1016/j.xcrm.2024.101681.

ACKNOWLEDGMENTS

H.C.A. and C.D. would like to acknowledge the Deutsche Forschungsgemeinschaft (DFG, German Research Foundation) for funding this work (grant numbers 404478562 and 446617142). The TARGET study was funded by the Federal Ministry of Education and Research (BMBF), 01EO1502, and TARGET Trial Investigators: Friedhelm Bach, Thorsten Brenner, Hendrik Bracht, Alexander Brinkmann, Thorsten Annecke, Andreas Hohn, Markus Weigand, Guido Michels, Stefan Kluge, Axel Nierhaus, Dominik Jarczak, Christina König, Dirk Weismann, Otto Frey, Dominic Witzke, Carsten Müller, Michael Bauer, Michael Kiehntopf, Sophie Neugebauer, Thomas Lehmann, Jason A. Roberts, and Mathias W. Pletz on behalf of the TARGET Trial Investigators.

AUTHOR CONTRIBUTIONS

Conceptualization, H.C.A. and C.A.; methodology, H.C.A. and C.A.; investigation, C.A. and A.A.; writing – original draft, H.C.A. and C.A.; writing – review and editing, H.C.A., C.A., C.D., S.H., M.O.C., and J.A.R.; funding acquisition, C.D. and C.A.; resources, C.A. and S.H.; supervision, C.A., C.D., and S.H.

DECLARATION OF INTERESTS

The authors declare no competing interests.

Received: November 13, 2023
Revised: May 25, 2024
Accepted: July 18, 2024
Published: August 9, 2024

REFERENCES

1. Williams, P.G., Tabah, A., Cotta, M.O., Sandaradura, I., Kanji, S., Scheetz, M.H., Imani, S., Elhadi, M., Luque-Pardos, S., Schellack, N., et al. (2023). International survey of antibiotic dosing and monitoring in adult intensive care units. Crit. Care *27*, 241–311. https://doi.org/10.1186/S13054-023-04527-1/TABLES/3.
2. Hagel, S., Bach, F., Brenner, T., Bracht, H., Brinkmann, A., Annecke, T., Hohn, A., Weigand, M., Michels, G., Kluge, S., et al. (2022). Effect of therapeutic drug monitoring-based dose optimization of piperacillin/tazobactam on sepsis-related organ dysfunction in patients with sepsis: a randomized controlled trial. Intensive Care Med. *48*, 311–321.
3. Jager, N.G.L., van Hest, R.M., Lipman, J., Taccone, F.S., and Roberts, J.A. (2016). Therapeutic drug monitoring of anti-infective agents in critically ill patients. Expet Rev. Clin. Pharmacol. *9*, 961–979. https://doi.org/10.1586/17512433.2016.1172209.
4. Adnan, S., Li, J.X., Wallis, S.C., Rudd, M., Jarrett, P., Paterson, D.L., Lipman, J., Udy, A.A., and Roberts, J.A. (2013). Pharmacokinetics of meropenem and piperacillin in critically ill patients with indwelling surgical drains. Int. J. Antimicrob. Agents *42*, 90–93. https://doi.org/10.1016/j.ijantimicag.2013.02.023.
5. Roberts, J.A., Ulldemolins, M., Roberts, M.S., McWhinney, B., Ungerer, J., Paterson, D.L., and Lipman, J. (2010). Therapeutic drug monitoring of β-lactams in critically ill patients: Proof of concept. Int. J. Antimicrob. Agents *36*, 332–339. https://doi.org/10.1016/j.ijantimicag.2010.06.008.
6. Roberts, J.A., Paul, S.K., Akova, M., Bassetti, M., De Waele, J.J., Dimopoulos, G., Kaukonen, K.M., Koulenti, D., Martin, C., Montravers, P., et al. (2014). DALI: defining antibiotic levels in intensive care unit patients: are current β-lactam antibiotic doses sufficient for critically ill patients? Clin. Infect. Dis. *58*, 1072–1083. https://doi.org/10.1093/CID/CIU027.
7. Gonçalves-Pereira, J., and Póvoa, P. (2011). Antibiotics in critically ill patients: A systematic review of the pharmacokinetics of β-lactams. Crit. Care *15*, R206. https://doi.org/10.1186/cc10441.
8. Ates, H.C., Roberts, J.A., Lipman, J., Cass, A.E.G., Urban, G.A., and Dincer, C. (2020). On-Site Therapeutic Drug Monitoring. Trends Biotechnol. *38*, 1262–1277. https://doi.org/10.1016/j.tibtech.2020.03.001.

9. Legg, A., Carmichael, S., Chai, M.G., Roberts, J.A., and Cotta, M.O. (2023). Beta-Lactam Dose Optimisation in the Intensive Care Unit: Targets, Therapeutic Drug Monitoring and Toxicity. Antibiotics *12*, 870. https://doi.org/10.3390/antibiotics12050870.
10. Abdulla, A., Ewoldt, T.M.J., Hunfeld, N.G.M., Muller, A.E., Rietdijk, W.J.R., Polinder, S., van Gelder, T., Endeman, H., and Koch, B.C.P. (2020). The effect of therapeutic drug monitoring of beta-lactam and fluoroquinolones on clinical outcome in critically ill patients: The DOLPHIN trial protocol of a multi-centre randomised controlled trial. BMC Infect. Dis. *20*, 57. https://doi.org/10.1186/s12879-020-4781-x.
11. Hagel, S., Fiedler, S., Hohn, A., Brinkmann, A., Frey, O.R., Hoyer, H., Schlattmann, P., Kiehntopf, M., Roberts, J.A., and Pletz, M.W.; TARGET Study Group (2019). Therapeutic drug monitoring-based dose optimisation of piperacillin/tazobactam to improve outcome in patients with sepsis (TARGET): a prospective, multi-centre, randomised controlled trial. Trials *20*, 330–410. https://doi.org/10.1186/s13063-019-3437-x.
12. De Waele, J.J., Carrette, S., Carlier, M., Stove, V., Boelens, J., Claeys, G., Leroux-Roels, I., Hoste, E., Depuydt, P., Decruyenaere, J., and Verstraete, A.G. (2014). Therapeutic drug monitoring-based dose optimisation of piperacillin and meropenem: a randomised controlled trial. Intensive Care Med. *40*, 380–387. https://doi.org/10.1007/S00134-013-3187-2.
13. Sime, F.B., Roberts, M.S., Tiong, I.S., Gardner, J.H., Lehman, S., Peake, S.L., Hahn, U., Warner, M.S., and Roberts, J.A. (2015). Can therapeutic drug monitoring optimize exposure to piperacillin in febrile neutropenic patients with haematological malignancies? A randomized controlled trial. J. Antimicrob. Chemother. *70*, 2369–2375. https://doi.org/10.1093/JAC/DKV123.
14. Mouton, J.W., Vinks, A.A., Felton, T.W., Schentag, J.J., Drusano, G., and Frey, O.R. (2015). Challenges and Potential Solutions - Individualised Antibiotic Dosing at the Bedside for Critically Ill Patients: a structured review. Lancent Infect Dis *14*, 498–509. https://doi.org/10.1016/S1473-3099(14)70036-2.Challenges.
15. Brasier, N., Ates, H.C., Sempionatto, J.R., Cotta, M.O., Widmer, A.F., Eckstein, J., Goldhahn, J., Roberts, J.A., Gao, W., and Dincer, C. (2023). A three-level model for therapeutic drug monitoring of antimicrobials at the site of infection. Lancet Infect. Dis. *23*, e445–e453. https://doi.org/10.1016/S1473-3099(23)00215-3.
16. Wicha, S.G., Märtson, A.G., Nielsen, E.I., Koch, B.C.P., Friberg, L.E., Alffenaar, J.W., and Minichmayr, I.K. (2021). From Therapeutic Drug Monitoring to Model-Informed Precision Dosing for Antibiotics. Clinical Pharmacology \& Therapeutics Preprint at John Wiley and Sons Inc *109*, 928–941. https://doi.org/10.1002/cpt.2202.
17. Ates, H.C., Mohsenin, H., Wenzel, C., Glatz, R.T., Wagner, H.J., Bruch, R., Hoefflin, N., Spassov, S., Streicher, L., Lozano-Zahonero, S., et al. (2022). Biosensor-Enabled Multiplexed On-Site Therapeutic Drug Monitoring of Antibiotics. Adv. Mater. *34*, 2104555. https://doi.org/10.1002/adma.202104555.
18. Rawson, T.M., Wilson, R.C., O'Hare, D., Herrero, P., Kambugu, A., Lamorde, M., Ellington, M., Georgiou, P., Cass, A., Hope, W.W., et al. (2021). Optimizing antimicrobial use: challenges, advances and opportunities. Nature Reviews Microbiology *19*, 747–758. https://doi.org/10.1038/s41579-021-00578-9.
19. Herrero, P., Wilson, R.C., Armiger, R., Roberts, J.A., Holmes, A., Georgiou, P., and Rawson, T.M. (2022). Closed-loop control of continuous piperacillin delivery: An in silico study. Front. Bioeng. Biotechnol. *10*, 1985. https://doi.org/10.3389/FBIOE.2022.1015389/BIBTEX.
20. Gowers, S.A.N., Freeman, D.M.E., Rawson, T.M., Rogers, M.L., Wilson, R.C., Holmes, A.H., Cass, A.E., and O'Hare, D. (2019). Development of a Minimally Invasive Microneedle-Based Sensor for Continuous Monitoring of β-Lactam Antibiotic Concentrations in Vivo. ACS Sens. *4*, 1072–1080. https://doi.org/10.1021/acssensors.9b00288.
21. Rawson, T.M., Gowers, S.A.N., Freeman, D.M.E., Wilson, R.C., Sharma, S., Gilchrist, M., MacGowan, A., Lovering, A., Bayliss, M., Kyriakides, M., et al. (2019). Microneedle biosensors for real-time, minimally invasive drug monitoring of phenoxymethylpenicillin: a first-in-human evaluation in healthy volunteers. Lancet. Digit. Health *1*, e335–e343. https://doi.org/10.1016/S2589-7500(19)30131-1.
22. Glatz, R.T., Ates, H.C., Mohsenin, H., Weber, W., and Dincer, C. (2022). Designing electrochemical microfluidic multiplexed biosensors for on-site applications. Anal. Bioanal. Chem. *414*, 6531–6540. https://doi.org/10.1007/s00216-022-04210-4.
23. Johnston, M., Ceren Ates, H., Glatz, R.T., Mohsenin, H., Schmachtenberg, R., Göppert, N., Huzly, D., Urban, G.A., Weber, W., and Dincer, C. (2022). Multiplexed biosensor for point-of-care COVID-19 monitoring: CRISPR-powered unamplified RNA diagnostics and protein-based therapeutic drug management. Mater. Today *61*, 129–138. https://doi.org/10.1016/j.mattod.2022.11.001.
24. Heil, E.L., Nicolau, D.P., Farkas, A., Roberts, J.A., and Thom, K.A. (2018). Pharmacodynamic target attainment for cefepime, meropenem, and piperacillin-tazobactam using a pharmacokinetic/pharmacodynamic-based dosing calculator in critically Ill patients. Antimicrob. Agents Chemother. *62*, e01008-18. https://doi.org/10.1128/AAC.01008-18.
25. Ates, H.C., Nguyen, P.Q., Gonzalez-Macia, L., Morales-Narváez, E., Güder, F., Collins, J.J., and Dincer, C. (2022). End-to-end design of wearable sensors. Nat. Rev. Mater. *7*, 887–907. https://doi.org/10.1038/s41578-022-00460-x.
26. Altay, A., Learney, R., Güder, F., and Dincer, C. (2022). Sensors in blockchain. Trends Biotechnol. *40*, 141–144. https://doi.org/10.1016/j.tibtech.2021.04.011.
27. Flores, M., Glusman, G., Brogaard, K., Price, N.D., and Hood, L. (2013). P4 medicine: How systems medicine will transform the healthcare sector and society. Personalized medicine *10*, 565–576. https://doi.org/10.2217/pme.13.57.
28. Virtanen, P., Gommers, R., Oliphant, T.E., Haberland, M., Reddy, T., Cournapeau, D., Burovski, E., Peterson, P., Weckesser, W., Bright, J., et al. (2020). SciPy 1.0: Fundamental Algorithms for Scientific Computing in Python. Nat. Methods *17*, 261–272. https://doi.org/10.1038/s41592-019-0686-2.
29. Reinhart, K., Brunkhorst, F.M., Bone, H.G., Bardutzky, J., Dempfle, C.E., Forst, H., Gastmeier, P., Gerlach, H., Gründling, M., John, S., et al. (2010). Prevention, diagnosis, therapy and follow-up care of sepsis: 1st revision of S-2k guidelines of the German sepsis society (Deutsche Sepsis-Gesellschaft e.V. (DSG)) and the German interdisciplinary association of intensive care and emergency medicine (Deutsche Interdisziplinäre Vereinigung für Intensiv- und notfallmedizin (DIVI)). Ger. Med. Sci. *8*, Doc14. https://doi.org/10.3205/000103.
30. Kuhn, M., and Johnson, K. (2019). Feature Engineering and Selection: A Practical Approach for Predictive Models (CRC Press). https://doi.org/10.1201/9781315108230.
31. Fortin, F.-A., Rainville, F.-M.D., Gardner, M.-A., Parizeau, M., and Gagné, C. (2012). DEAP: Evolutionary Algorithms Made Easy. J. Mach. Learn. Res. *13*, 2171–2175.
32. Pedregosa, F., Varoquaux, G., Gramfort, A., Michel, V., Thirion, B., Grisel, O., Blondel, M., Prettenhofer, P., Weiss, R., Dubourg, V., et al. (2011). Scikit-learn: Machine Learning in Python. J. Mach. Learn. Res. *12*, 2825–2830.
33. Mitchell, M. (1999). An Introduction to Genetic Algorithms (The MIT Press).

STAR★METHODS

KEY RESOURCES TABLE

REAGENT or RESOURCE	SOURCE	IDENTIFIER
Deposited data		
Patient data	Hagel et al.[2]<br>Hagel et al.[11]	German Clinical Trials Register (GermanCTR), DRKS00011159
Software and algorithms		
scikit-learn	https://pypi.org/project/scikit-learn/	N/A
deap	https://pypi.org/project/deap/	N/A
sklearn-genetic	https://pypi.org/project/sklearn-genetic/	N/A
statsmodels	https://pypi.org/project/statsmodels/	N/A

RESOURCE AVAILABILITY

Lead contact

Further information and requests for resources should be directed to the lead contact, Dr. Can Dincer (dincer@imtek.de).

Materials availability

This study did not generate new unique reagents.

Data and code availability

- All data processing and modeling were conducted on Python 3 using standard libraries that are publicly available: *pandas, numpy, scipy, scikit-learn, matplotlib, seaborn, plotly, category-encoders, deap, sklearn-genetic, statsmodels.* The code utilized in this study was tailored to the data collected in the clinical study[2] and its unique data structure. Without access to the data, the code holds little utility; however, interested readers may request access from the lead contact.
- This paper analyses existing data collected in the clinical study and can be accessed from an already published open-access publication.[2]
- Any additional information required to reanalyse the data reported in this paper is available from the lead contact upon request.

EXPERIMENTAL MODEL AND STUDY PARTICIPANT DETAILS

Study design and objectives

The data source used in this work had been published as a randomized, controlled trial, which involved patients admitted with severe sepsis or septic shock and aimed to compare the clinical effectiveness of TDM-guided Piperacillin/Tazobactam antibiotic therapy versus a fixed dosing strategy.[2] The working hypothesis here is that the information regarding the recovery process is embedded into the measured features. In other words, healthier states should be distinguishable from relatively sick states by comparing informative measured features at different times for a patient, or in between patients. Such an approach converts the problem of quantifying the effect of TDM into a state trajectory analysis; that is, via monitoring the change in these features, recovery rate can be measured in the state space. More importantly, it becomes possible to quantify relative recovery rates with or without TDM, as a more effective therapy will change the recovery trajectory for the patient.

Details of the clinical study

A clinical trial was conducted to compare the effectiveness of TDM-guided antibiotic therapy with fixed dosing in improving clinical outcomes in sepsis patients treated with piperacillin/tazobactam.[2] The trial included 248 adult patients with severe sepsis or septic shock who had received the therapy within the last 24 h before enrollment. It took place in 13 different locations in Germany between January 2017 and December 2019 and was randomized, controlled, and patient blinded. The trial was registered at the German Clinical Trials Register (GermanCTR), DRKS00011159, as required by the funding agency. All details of the trial can be accessed via https://drks.de/search/de/trial/DRKS00011159.

The study recorded clinical, microbiological, and laboratory data from the day prior to randomization and then throughout the following time points: day 14 post randomization, at the end of therapy, at discharge from the ICU, and at day 28. Patients were randomly assigned (1:1) to either the TDM group or to the control group (no-TDM). Randomization was stratified by the participating centers and performed by the investigators using an internet-based randomization tool. Following randomization, both the control

and TDM groups were given an initial loading dose of 4.5 g of piperacillin/tazobactam, followed by a continuous infusion of the same antibiotic. The total daily dose was 13.5 g (9 g in patients with an estimated glomerular filtration rate (eGFR) < 20 mL min^{-1}). In the TDM group, dosing of piperacillin/tazobactam was guided by daily monitoring of piperacillin, starting on Day 1 post randomization (or Day 0 if the piperacillin concentration had already reached a steady state) for a maximum of 10 days. Use of antimicrobial combination therapy, termination or (de-) escalation of antimicrobial therapy was allowed at any time and at the discretion of the treating physicians. The target plasma concentration of free piperacillin was set to four times (with a range of ±20%) the minimal inhibitory concentration of the pathogen responsible for sepsis. For empirical therapy, the epidemiological cut-off (ECOFF) of Pseudomonas aeruginosa (16 mg L^{-1}) published by the European Committee on Antimicrobial Susceptibility Testing (EUCAST) was utilized. In patients receiving TDM-guided therapy with piperacillin/tazobactam, a dose adjustment was made on 53.9% (312/579) of the treatment days. In the control group, daily dose adjustments were based on patient renal function and did not utilize any TDM. Both patient cohorts had blood samples taken daily to measure piperacillin concentrations. The TDM group received same-day analysis, reporting, and dose adjustments, while analysis in the control group could be performed on the same day or later, with samples kept at −80°C until analyzed. Total piperacillin concentration measurements were performed on-site in study centers using either high-performance liquid chromatography (HPLC) or liquid chromatography mass spectrometry (LC-MS/MS). The trial protocol was approved by institutional review boards, published previously, and Germany's Federal Institute for Drugs and Medical Devices (EudraCT: 2016-000136-17, ref. 4041358). The primary endpoint was sepsis-related organ dysfunction measured by the mean daily total SOFA scores over 10 days, discharge from the ICU or death, whichever occurred first. The mean SOFA score was calculated as the mean of all daily SOFA scores for each patient.

It should also be noted that sepsis was defined according to the criteria valid at the time of initiation of the study (Sepsis-2 criteria). It should also be noted that sepsis was defined according to the criteria valid at the time of initiation of the study (Sepsis-2 criteria).[29] At the time the study was planned and initiated, the old sepsis definitions were still valid. The inclusion criteria were not changed during the ongoing study to comply with the new Sepsis-3 definition. Interested readers are encouraged to refer to the original publication for further details of the clinical study.[2]

METHOD DETAILS

Patient state and measure of similarity

Mahalanobis distance is a statistical measure used to assess the dissimilarity between a sample point and a distribution in a multidimensional space, considering the structure of the data. The Mahalanobis distance from a patient state vector A to a reference distribution R with mean μ and covariance Σ is calculated as:

$$D(A,R) = \left((A-\mu)\Sigma^{-1}(A-\mu)^T\right)^{1/2}$$

where Σ^{-1} is the inverse of the covariance matrix of the reference distribution R. The Mahalanobis distance accounts for the correlation between different measured variables by scaling the differences with the inverse covariance matrix.[28] Considering the inter-patient variance in the measured physiologically relevant features, it is considered that the Mahalanobis distance would be the best fit to mathematically describe the dissimilarities between the patient states.

In particular, we used the Mahalanobis distance to measure dissimilarity between the patient state at a given time and a reference "healthy state distribution" based on the SOFA score. Firstly, we investigated the uniqueness of the SOFA scores during the Exploratory Data Analysis (EDA) phase, revealing that only six patient states have a score of SOFA = 0. As a result, second best SOFA score, SOFA = 1 is used as a filter to create a state vector group as the reference distribution. Figure 1C depicts the measurement of Mahalanobis distances for each patient state at each day in a 2D feature space. Herein, blue and orange points mark the state vectors of the patients in a 2D state space for the TDM and control groups, respectively. In both groups, patients start their recovery trajectory at a certain sub-space of the 2D state space. The distance to the reference health state (for example, d_1) is expected to be correlated with the degree of dissimilarity with the state of "being healthy". As the therapy continues successfully, the patient should "move" in the feature space toward the reference state and the rate of recovery is correlated with how fast the groups move from their initial states (day of admission) to the reference zone, so called "healthy town". In other words, if the dose adjustment within TDM is beneficial for the TDM group, there must be a distinct difference between how much closer they are to the healthy zone compared to the control group. This is quantified by calculating the cumulative sum of the Mahalanobis distance between the TDM/control groups and the reference states for each day (t):

$$CumSum(t) = \sum_{p=1}^{P(t)} D_{\text{Mahalanobis } p}(t)$$

Where p is the patient for which the distance ($D_{\text{Mahalanobis } p}$) is being calculated for day t and $P(t)$ the number of patients that are in the TDM/control group at day t. To ensure the statistical significance of the measured CumSum, daily values are normalized based on the mean pairwise distance of SOFA = 1. In other words, normalized distances shown in Figure 2C report how far away the TDM/control group to the reference state, if the distance between SOFA = 1 patients is equal to 1.

It should be also noted here that since the feature set is heterogeneous (i.e., consists of continuous, categorical and ordinal variables), discrete ones should be first transformed into a pseudo-continuous representation, and then all features should be scaled for an unbiased dissimilarity analysis. In this work, the features are first grouped into three sub-sets: continuous, discrete and discrete pathogen-related features (see Figure S2). Then, discrete features are transformed via CatBoost[30] to have a continuous feature space. Next, all features are normalized with standard scaling before conducting the distance analysis. CatBoost and standard scaling methods are fit by using only the training data to prevent data leakage.

Feature engineering and selection

Mathematical similarity, which is type of unsupervised learning approach, relies on the measured distances in high dimensional feature space, which makes feature engineering and selection very critical, particularly for the conducted analysis with limited number of patients and total number of daily observations (state vectors).[30] In the current study, the primary clinical data is first analyzed in terms of feature variance, missing data, outliers, and any other unphysical abnormalities (see Figure S1). The data preparation steps (see Figure S2) converted primary clinical data into 199 structured features for 248 patients (TDM:123, control:125) with variable trajectory lengths (i.e., duration of the treatment at daily granularity), consisting of 2376 state vectors in total. Since the clinical data is limited, high dimensional feature space (199 dimensions) is found to be extremely sparse, the dimensionality of the problem has to be reduced, as the similarity analysis relies on distance, and the “signal/noise ratio” in distance-based (or error-based) approximations diminishes exponentially as the number of features increases.[30] In the context of conducting multidimensional SOFA score analysis through distance calculations within the latent space, concerns about interpretability can arise, particularly when utilized by scientists and clinicians from diverse backgrounds. There is a risk that such projections would merge different spatial and/or temporal features, making the analysis a black box model. To mitigate these concerns and enhance interpretability, feature selection was employed to simplify the feature space prior to conducting distance calculations. This was also one of the reasons why we did not rely on neural network-based dimensionality reduction techniques. Therefore, in the next step, we applied alternative feature selection approaches including filtering, implicit and wrapper methods. For this particular problem, using genetic algorithms (GA) for feature selection[31,33] (i.e., as a wrapper) provided the best feature subset, and the methodology for feature selection is described here only for the GA implementation.

Firstly, we leave 10 patients out of the feature selection study to increase and test the generalizability of the similarity approach. The feature selection procedure is shown in Figure 1B. The process starts with a train and test split for the selected 238 patients. The training data is then passed to the Wrapper, which is a GA implementation with internal cross-validation (CV). A random forest model[32] was used as the estimator of the feature selection wrapper. The score needed to iteratively refine the feature subset is taken as the SOFA scores, where the metric for the fitness is selected as the negative mean absolute error. The number of feature subset is also scanned parametrically, starting from one to the maximum number of features to analyze the value of added information with increased number of features. The CV scores are then examined to determine the optimum number of features. As the GA involves randomness, feature selection process is repeated 100 times, where 100 generations are created at each run. The frequency of the features selected by the last GA generations is given in Figure 4. It is seen that pathogen related data was rarely selected by the model, typically less than 10% of the time. For the final set, the features that were picked more than 10% of the time were unionized with a new GA iteration to cover potential multivariate correlations (Figure 4, dark blue). During the preliminary studies, various shallow ML predictors with the feature selection loop were also evaluated. An ensemble, information-based random forest model demonstrated the best generalizability and highest performance on test data, as well as for the patients left out of the analysis. More details about the feature selection scores are presented in the Supplementary Information (see Figures S1–S3).

SOFA score and patient analysis

The cumulative Mahalanobis distance analysis described in the previous section provides a way to quantify the impact of TDM on the patient status in the form of mathematical dissimilarity between the current state and a reference distribution. To further analyze how the abstract distances translate into patient recovery, we examined the (i) temporal evolution of the patient SOFA scores, and (ii) mortality rate for both TDM and control groups.

The SOFA score is a clinical tool utilized to evaluate the severity of illness and prognosis in critically ill patients. It involves assessing six organ systems: respiratory, cardiovascular, hepatic, coagulation, renal, and neurological. Each organ system is assigned a score ranging from 0 to 4, where higher scores indicate more severe dysfunction. The individual scores for each organ system are summed to obtain a total SOFA score, which can range from 0 to 24. A higher score indicates more pronounced organ dysfunction and a poorer prognosis for the patient. In the current study, SOFA scores are used in the supervised ML methods for feature selection, and to interpret and discuss the calculated Mahalanobis distances. We also conducted statistical analysis on the distribution of patient health status by examining the multivariate feature distributions on the day of admission, as well as on the last recorded patient data (state vectors). Patient analysis includes the comparison of the first day state vectors of the patients to justify the controlled clinical trial with dimensionality reduction techniques qualitatively and the Mahalanobis distances quantitatively. Pathogen tests of the TDM and control groups are also compared for the first day to ensure that TDM and control split of the patients is not biased toward any group. In other words, the pathogen distributions and their piperacillin resistances should also be distributed in a balanced way between the TDM and control group patients. Furthermore, for each day of the therapy, medians of the last recorded SOFA score for alive patients and the day patients leave the clinical study are extracted from the log files to discuss the results obtained by the similarity-based state analysis.

QUANTIFICATION AND STATISTICAL ANALYSIS

Kolmogorov-Smirnov (KS) test

When applied to a cumulative distribution function (CDF), KS test serves as a measure of dissimilarity between two distributions. Given $F(x)$ representing a theoretical CDF, and a sample of n observations from an empirical distribution function $F_n(x)$, the Kolmogorov-Smirnov test statistic D_{KS} is defined as:

$$D_{KS} = max(|F_n(x) - F(x)|)$$

D_{KS} yields the supremum of the absolute differences between the two distributions over the entire range of possible values, which quantifies how well the empirical distribution function fits the hypothesized theoretical distribution. If D_{KS} is small, the two distributions are considered to be similar, otherwise it indicates a significant dissimilarity between the empirical and theoretical distributions. In this study, it is used to compare two cumulative distribution functions (CDFs), namely the TDM and control group data. In such a scenario, the test assesses whether the two distributions differ significantly or not. Herein, the null hypothesis (H_0) states that the two distributions are two distributions, while the alternative hypothesis (H_1) claims the opposite.

Hellinger distance

This is another measure of the similarity between two probability density functions (PDFs). The Hellinger distance D_H between these two PDFs $f(x)$ and $g(x)$ is defined as:

$$D_H(f,g) = \sqrt{0.5 \int \left(\sqrt{f(x)} - \sqrt{g(x)}\right)^2 dx}$$

Similarly, a large D_H value suggests greater dissimilarity between the two distributions. It is worth to note that D_H is bounded between 0 and 1. The threshold value is typically based on the context of the problem.

Multiple factor analysis of variance

Multi-factor analysis of variance (ANOVA) is a statistical method used to examine whether the mean levels of various effects are equal across multiple factors or conditions simultaneously. This analysis typically involves a response variable (dependent) and one or more factor variables (independent). Each factor can have different levels, representing distinct conditions or categories. In this study, two factors were investigated: 'Group' (including TDM or Control) and 'Time' (indicating treatment day). The dependent variable of interest was the last recorded SOFA score for the patients left the study alive. By regressing the SOFA score against both 'Group' and 'Time', the individual contributions of these factors to variations in the dependent variable were assessed. The model fitting employed Ordinary Least Squares (OLS) regression. Specifically, Type 2 ANOVA, also known as sequential ANOVA, was utilized, which evaluates the significance of each independent variable while adjusting for the effects of other independent variables in the model. Computational analyses were conducted using the statsmodels library.

ADDITIONAL RESOURCES

The data used in this study is based on an already published clinical trial under German Clinical Trials Register (GermanCTR), DRKS00011159.

Data preparation procedure

There were 12 files constituting the primary clinical data. In each, the patient data is encoded as follows:
rno: Ta-01-0001-3.

(1) 01: which site/hospital
(2) 0001: patient number

enabling to filter and process patient specific features, and create virtual patient cards. The content of each data is summarized below:

dm: Demographic information. It includes features like age, gender, height, body weight, hospital admission date, ICU admission date, type of assignment, where before ICU.
vs.: Physiological data such as PF Ratio (paO2/FiO2 ratio; only arterial measurement, lowest paO2 +associated FiO2)).
pip: Piperacillin therapy related data.
lb: Lab measurements are reported in this document.
mibi: Microbiology lab results.
sep: Sepsis related file. It contains information about site of infection, if sepsis happened and response to drug.
amic: Antimicrobial therapy related data.
amyk: Antifungal treatment related data.

rand: Tells which patient gets the TDM and which one is the control group.

chir: Surgical drainage data.

ccmc: Clinical cure data. Most important column is the clinical cure. The rest is considered as supplementary data.

com: Additional comments for some patients. Not used in this work.

Data preparation pipeline starts with data cleaning. All primary files are treated individually to exclude uninformative, redundant features. Furthermore, if there is more than one entry for a feature at a given day, they are filtered and saved as a list at the first step. In other words, each patient has one entry for each feature, at every day where the length of the entry may vary from patient to patient, feature to feature, or day to day. In the first iteration, all information filtered is kept as lists of entries for each day. Below, the decision made during the data cleaning is summarized.

(1) For any feature, in any file, if the values are saved with different units, they are all converted into the same unit (e.g., if majority is reported as mg, all reported as mg and g information is converted to mg).
(2) A new "dead" feature is created. The entry implying that patient is dead or in palliation (['Tod', 'Patient verstorben', 'verstorben', 'Versterben', 'Pat. verstorben', 'Tod des Patienten', 'Palliation, Exitus', 'Palliation', 'Death', 'Todesfall']) was not consistent and thus, we used filters to assign numerical values of 0 and 1 for alive and dead patients.
(3) underscoreX: Some features (e.g., START_X) denote whether corresponding feature has a missing value or not. As the information is already in the feature itself, underscore columns are dropped.
(4) ICU day: Since the patients are taken in ICU shortly after hospital admission, the feature had little variance in between patients, and dropped accordingly.
(5) Zero variance features are dropped, such as "zksno".
(6) We drop features with no information, or those repeat the information given in the other features: where_before_ICU, site, time2, dsno, KONZENT_X new_dosage_day, VISITE_D_new, START_D_new,START_Z_new, STOPP_D_new, STOPP_Z_new, KONZENT_Z_new, STOPP_Z_new, LARATENEU_D_new, LARATENEU_Z_new UNIT_C (after unit conversions), WISTOFF_E, WISTOFF_C, ANTIMYK_B, hearthrate_NA, PF_unit(1:kPa-2:mmHg), new_dosage_day, comment_why_pip_changed, pip_mic_na, other_pathogen, 'Randomization_day', 'Randomization_time', 'RANDOMNR_C'
(7) We also remove rows with ANTIMYK_B = = 1, as it contained no information.
(8) From the clinical cure data, only the columns 'Clinical_cure(1: healing-2:improvement-3:failure-9:na)', 'Microbio_cure(1=>7–9)' are kept.

At the next step, the clean 11 data files that are saved in a different directory is read and combined under a single pandas dataframe.

Next, we compute the length of each cell in the df to decide the time granularity of the state space analysis. Extracting the lengths of features for each patient and for each day revealed that the study can be conducted only at daily granularity. As a result, features that are recorded multiple times in some patients (in some days) are further processed.

(1) Columns that contain no information are dropped.
(2) For missing values (less than 5% column wise), numerical features are imputed with the column median value, while categorical values are filled with the most frequent one.
(3) For the following features, if there is more than one measurement, daily average is used to represent the patient state:
'heathrate_min', 'heathrate_max', 'mean_art.bloodpressure_min', 'mean_art.bloodpressure_max',
'temp_min', 'temp_max', 'breathing_rate_min', 'breathing_rate_max', 'ph_min', 'ph_max',
'PF_ratio_min', 'pO2_at_lowest_PF_ratio', 'FIO2_at_lowest_PF_ratio', 'urine_output(mL)',
'urine_collection(h)', 'kreatinin_min', 'crp_max', 'pct_max', 'leukocytes_min', 'leukocytes_max',
'thrombocit_min', 'pip_dosage(mg)', 'kreatinin_max', 'contd_inf_rate(mL/h)', 'sample_conc(mg/L)',
'new_dosage(mL/h)', 'bicarbonate_min', 'bicarbonate_max', 'pip_dosage(mg).1', 'target_conc(mg/L)'
(4) Features that include explanations or notes taken during the clinical study is dropped, as there were not sufficient examples for text mining:

'Stop_reason(why_8)', 'STARTGRUND_C', 'STOPPGRUND_C', 'reason'

(5) Columns start with the following is dropped as they contain repeated time information:

'rno', 'STARTZ_Z', 'STOPPZ_Z', 'STOPP_D', 'Stop_time', 'Visite_', 'day', 'hour', 'Date', 'date', 'KONZENT_D_new'

(6) Following columns are reorganized, before one hot encoding:

'type_of_ventilation(0:*n*-1:noninvasive-2:invasive)', 'renal_replacement(0:*n*-1:y)', 'Pneumo(0:*n*-1:y)', 'Trach_secration(0:little-1:abundant-2:ab_with_prulent)', 'Infiltrate(0:*n*-1:diffuse-2:localized)', 'ARDS(0:*n*-1:y)',

'eye_response(4:spontaneous-3:after_prompt-2:on_pain_sti-1:no)', 'Verbal(5:clear-4:confused-3:single_words-2:single_sounds-1:no)',

'Motor(6=>1_getting_worse)', 'GCS(1:raised-2:estimated)', 'Drainage(0:*n*-1:y)', 'pip_give(0:*n*-1:y)', 'pip_give_type(1:empric-2:targetted)', 'Bolus_delivery(0:*n*-1:y)', 'blood_sampling(0:*n*-1:y)', 'Drainage_number', 'new_dosage_reason(1:TDM-2:side-effect-3:other)',

'Clinical_cure(1:healing-2:improvement-3:failure-9:na)', 'Microbio_cure(1=>7–9)', 'change_in_pip(0:*n*-1:y)', 'why_therapy_stop(1=>8)', 'CHECK1_B(0:*n*-1:y)', 'CHECK2_B′, 'CHECK2_E′, 'CHECK3_B′, 'CHECK3_E′, 'CHECK4_B′, 'CHECK4_E′, 'Pathogen_type(unique_values)', 'can_cause_sepsis(0:unlikely-1:probable-9:unknown)', 'pip_resistance(1:sensitive-2:intermediate-3:resistant-9:not_tested)', 'pip_mic(mg/l)', 'target_based_on(0:no_patogen-1:patogen)'

(7) Columns with too many NaN values are dropped:

'bicarbonate_v_min', 'bicarbonate_v_max', 'APPLI_E′, 'GESDOSIS_N′, 'Antimic(0:*n*-1:y)', 'CHECK1_E(day)',
'CHECK2_E′, 'CHECK3_E′, 'CHECK4_E′, 'pip_mic(mg/l)'

(8) Columns related to time information were further filtered, together with meta columns:

'Sepsis_day', 'Sepsis_hour', 'reason', 'pip_start_hour', 'pip_stop_hour'
'Visite_day_pip_clean_reshape', 'Visite_day_sep_clean_reshape',
'Start_day_pip_clean_reshape', 'Start_hour_pip_clean_reshape', 'START_D′, 'STARTZ_Z′, 'STARTGRUND_E′, ' STOPP_D′, 'STOPPZ_Z′, 'STOPPGRUND_E′, 'STOPPGRUND_C′, 'Visite_day_ccmc_clean_reshape', 'site', 'Visite_day_chir_clean_reshape', 'Date_micobio_results', 'Start_day', 'Start_hour', 'Start_reason(1=>6)', 'STARTGRUND_C′, 'Stop_day', 'Stop_time', 'Stop_reason(1=>8)', 'Stop_reason(why_8)', 'blood_sampling_day', 'blood_sampling_hour', 'why_therapy_stop(1=>8)', 'Stop_pip_day', 'Stop_pip_hour', 'new_dosage_hour', 'new_dosage_reason(1:TDM-2:side-effect-3:other)', 'KONZENT_D_new', 'day_visited', 'hospital_admission_date', 'hospital_admission_hour', 'ICU_date', 'ICU_hour', 'type_of_assigment', 'where_before_ICU'

The data cleaning process conducted reduced the number of base features to 100, and number of patients to 253 so far.

Pathogen related information is then encoded. Herein, we created binarized features for each pathogen type, whether they cause sepsis and whether that particular pathogen has pip resistance or not. For that purpose, we filter the pathogen data:
['Pathogen_type(unique_values)', 'can_cause_sepsis(0: unlikely-1:probable-9:unknown)',
'pip_resistance(1:sensitive-2:intermediate-3:resistant-9:not_tested)'] where pathogen type can take values of:
101, 102, 103, 104, 106, 107, 108, 110, 111, 201, 202, 203, 204, 205, 206, 207, 208, 209, 210, 211, 212, 301, 302, 303, 305, 501, 502, 503, 504, 505, 506, 507, 508, 509, 1051,1052]

After data encoding, pathogen information is binarized:

Pathogen_101(0:n-1:y)	Pathogen_102(0:n-1:y)	Pathogen_103(0:n-1:y)
0	0	0
0	0	0
0	0	0
0	0	0
0	0	0

with corresponding resistance and sepsis information:

pip_resistance_508(1:sensitive-2:intermediate-3:resistant-9:not_tested)	pip_resistance_509(1:sensitive-2:intermediate-3:resistant-9:not_tested)	pip_resistance_1051(1:sensitive-2:intermediate-3:resistant-9:not_tested)
0	0	0
0	0	0
0	0	0
0	0	0
0	0	0

Finally, the remaining data frame is split as continuous and discrete, based on the number of unique elements in a column. If it is greater than 11, the column is considered to be continuous.

Pairwise distance analysis on day 1

After the data preparations, without any further dimensionality reduction/feature selection procedure, we checked the similarity between patients in Day 1 in the high dimensional feature space. This is done by computing the pairwise distance between patient i and all remaining patients, followed by an averaging. Figure S2A shows the mean Euclidian distance for each patient. Results revealed that the majority of the patients are at a similar distance to the rest of the patients, whether the patient is part of the control or TDM group. It should be noted that the distances are calculated after scaling, so as not to be biased with respect to large magnitude features.

Details of feature selection via wrapper

Wrapper methods employ iterative search procedures to select subsets of features for the model. These methods repeatedly provide feature subsets to the model and use the resulting model performance to guide the selection of the next subset for evaluation. The goal is to identify a smaller set of features that outperforms the original set-in terms of predictive performance. Wrapper methods offer the advantage of exploring a wider range of feature subsets than simple filters or models with built-in feature selection. However, the main drawback is the excessive computational time required to find the optimal or near optimal subset, particularly if the estimator is a computationally demanding model like neural networks. It should be also noted that wrappers can overfit to the training data, hence requires cross validation-based training procedures.

Wrapper methods can adopt either a greedy or non-greedy approach to feature selection. A greedy search chooses the search path based on the direction that seems the best at the current moment to achieve immediate benefits. While this strategy can be effective, it may reach a locally optimal setting where further improvements become difficult. In contrast, non-greedy search methods such as genetic algorithms (GA) re-evaluate previous feature combinations and have the flexibility to move in a direction that initially appears unfavourable but shows potential benefits in subsequent steps. This allows the non-greedy approach to avoid getting trapped in a local optimum, in which greedy search methods might got caught.

GAs employ a strategy inspired by natural evolution to effectively discover optimal solutions. They generate a set of candidate solutions for the optimization and allow them to reproduce and create new solutions using mating and mutations. Through competition, the most evolutionarily fit solutions, i.e., the optimal ones, have a greater likelihood of surviving and propagating into the next generation (natural selection). This iterative process enables genetic algorithms to gradually improve solutions over time and has demonstrated convergence for a diverse range of problems. For feature selection, the genetic material becomes the indices of feature columns of the original base dataframe, while the length of the genetic material defines the number of selected features. The fitness of a feature subset is calculated via an estimator. Herein, estimator solves a regression task: given the feature set, how accurate the model can predict the labels. In this work, SOFA scores are used as the label and the regression model is chosen to be either a distance-based linear model (multivariate linear regression, Lasso, ElasticNet), or information-based nonlinear models (Random Forests). In other words, we used supervised machine learning methods as the fitness function.

Considering the heterogeneous nature (i.e., continuous, categorical and ordinal features all together) of the patient data, we approached the feature selection with GA in 4 different ways based on (i) the estimator type being used (distance- or information-based) and (ii) whether the data is treated as a whole (199 features passed) or a sub-GA selection is conducted for the continuous, discrete and pathogenic features. For the former, as the distance-based supervised ML models require to work with scaled, continuous features, discrete data is first transformed with CatBoost, and then all features are scaled via a standard scalar. If random forest is used, features are kept as they are; that is, no transformation and no scaling. Secondly, as the dimensionality of the problem is large (199 features), it is considered worthy to investigate a “divide and conquer” approach: a different GA wrapper is used for continuous, discrete and pathogen features, to pick up informative features independently in lower dimensional space. Although lower

dimensionality helps to identify patterns in low data limit, this split assumes that continuous, discrete and pathogen feature selections do not have cross correlations. In accordance, we checked SOFA score prediction capabilities based on 4 scenarios.

(1) All features are passed to a single GA wrapper as they are (no CatBoost transformation, no scaling) with random forest estimator.
(2) Discrete features are transformed with CatBoost; then all features are scaled with standard scaler. Next, transformed and scaled features are passed to a single GA wrapper with a distance-based linear regression model.
(3) Feature set is split into three; continuous, discrete and discrete pathogen data. These 3 subsets of features are passed to 3 different GA wrapper as they are (no CatBoost transformation, no scaling) with random forest estimator.
(4) Feature set is split into three; continuous, discrete and discrete pathogen data. Discrete and discrete pathogen data are transformed with CatBoost independently; then all three subsets are scaled with standard scaler. Later, transformed and scaled subsets are passed to 3 different GA wrapper with its independent distance-based linear regression model.

For all cases, we first leave 10 patients out completely from the state space database, to leave of some patients for better generalizability. Then, we further conducted a 4:1 train-test split to ensure that feature selection procedure will not leak any information about the evaluation of the state space trajectory analysis. In the next step, we passed the train set of the patient states directly to the GA-based wrapper. Since we did not *a priori* how many features are enough to represent the patient state, we sweep through number features, starting from 1 to maximum number of features. For instance, in the case of *Feature Set A*, GA Wrapper is called 199 times, for number of features 1,2,3, …, 198, 199. For each number of features, (e.g., number_of_feature = 15), we initialize a population of 160 individuals, with randomly selected 15 features. Herein, the genes of an individual contain the indices of 15 randomly picked features. Then, for each individual, a different estimator is being trained with a cross validation scheme (cv = 5). The negative mean absolute error of that individual's cv scores is saved as the fitness. Then, the same is applied to all 160 individuals. This is followed by a parent selection, mating and mutation, which enables to update the gene pool. The whole GA process is applied for 100 generations, yielding the best feature sub-set (e.g., best 15 sub-features among 199). Lastly, the trained estimator with the best feature subset is used to generate repeated k-fold cv scores (RepeatedKFold(n_splits = 10, n_repeats = 3). Scores are saved. The process continues with the next number of features (e.g., number of features = 15 + 1 = 16) until the maximum number of features is reached.

Cell Reports Medicine, Volume *5*

Supplemental information

Unraveling the impact of therapeutic drug monitoring via machine learning for patients with sepsis

H. Ceren Ates, Abdallah Alshanawani, Stefan Hagel, Menino O. Cotta, Jason A. Roberts, Can Dincer, and Cihan Ates

rno	Visite_day	heathrate_min	heathrate_max	blood_pressure_min	blood_pressure_max	mean_art.bloodpressure_min	mean_art.bloodpressure_max	temp_min	...	Height	Weight
Ta-01-0001-3	['2017-01-26', '2017-01-27', '2017-01-28', '20...	['[46.0, 59.0]', '[56.0]', '[56.0]', '[46.0]',...	['[116.0, 113.0]', '[110.0]', '[86.0]', '[94.0...	['[63.0, nan]', nan, nan, nan, nan, nan, nan, ...	['[121.0, nan]', nan, nan, nan, nan, nan, nan,...	['[46.0, 64.0]', '[64.0]', '[69.0]', '[59.0]',...	['[86.0, 83.0]', '[81.0]', '[86.0]', '[81.0]',...	['[32.2, 34.6]', '[35.4]', '[35.7]', '[36.5]',...	...	170.0	60.0
Ta-01-0002-1	['2017-01-27', '2017-01-28', '2017-01-29', '20...	['[76.0, 80.0]', '[83.0]', '[72.0]', '[71.0]',...	['[115.0, 96.0]', '[129.0]', '[105.0]', '[95.0...	['[81.0, nan]', nan, nan, nan, nan, nan, nan]	['[122.0, nan]', nan, nan, nan, nan, nan, nan]	['[56.0, 65.0]', '[59.0]', '[58.0]', '[58.0]',...	['[77.0, 79.0]', '[82.0]', '[87.0]', '[83.0]',...	['[34.3, 37.1]', '[36.8]', '[36.9]', '[37.2]',...	...	164.0	60.0
Ta-01-0003-9	['2017-03-22', '2017-03-23', '2017-03-24', '20...	['[88.0, 94.0]', '[79.0]', '[76.0]', '[67.0]',...	['[174.0, 129.0]', '[112.0]', '[98.0]', '[80.0...	['[86.0, nan]', nan, nan, nan, nan, nan, nan, ...	['[149.0, nan]', nan, nan, nan, nan, nan, nan,...	['[62.0, 62.0]', '[60.0]', '[73.0]', '[79.0]',...	['[91.0, 112.0]', '[92.0]', '[107.0]', '[111.0...	['[36.9, 37.0]', '[36.8]', '[37.1]', '[37.2]',...	...	170.0	140.0
Ta-01-0004-6	['2017-03-24', '2017-03-25', '2017-03-26', '20...	['[67.0, 76.0]', '[58.0]', '[65.0]', '[81.0, n...	['[142.0, 118.0]', '[109.0]', '[105.0]', '[133...	['[79.0, nan]', nan, nan, nan, nan, nan, nan, ...	['[192.0, nan]', nan, nan, nan, nan, nan, nan,...	['[61.0, 61.0]', '[53.0]', '[59.0]', '[67.0, n...	['[133.0, 97.0]', '[102.0]', '[96.0]', '[114.0...	['[36.4, 37.1]', '[36.2]', '[37.7]', '[37.1, n...	...	185.0	80.0
Ta-01-0005-4	['2017-04-20', '2017-04-21', '2017-04-22', '20...	['[88.0, 84.0]', '[62.0]', '[60.0]', '[60.0]',...	['[178.0, 110.0]', '[87.0]', '[73.0]', '[89.0]...	['[78.0, nan]', nan, nan, nan, nan, nan, nan, ...	['[129.0, nan]', nan, nan, nan, nan, nan, nan,...	['[50.0, 56.0]', '[56.0]', '[56.0]', '[58.0]',...	['[76.0, 72.0]', '[84.0]', '[83.0]', '[106.0]'...	['[37.7, 36.5]', '[36.5]', '[36.4]', '[36.8]',...	...	155.0	55.0

Figure S1. Example of a master data frame for patient records, related to Fig. 1b-c. Only the first 11 features of 5 patients are shown in the table. Herein, each row represents a patient, and each column contains variable length lists of features. The shape of the data frame at this step was (254,152), denoting 152 features for 254 patients. First 11 column of the data is depicted below to demonstrate the df structure.

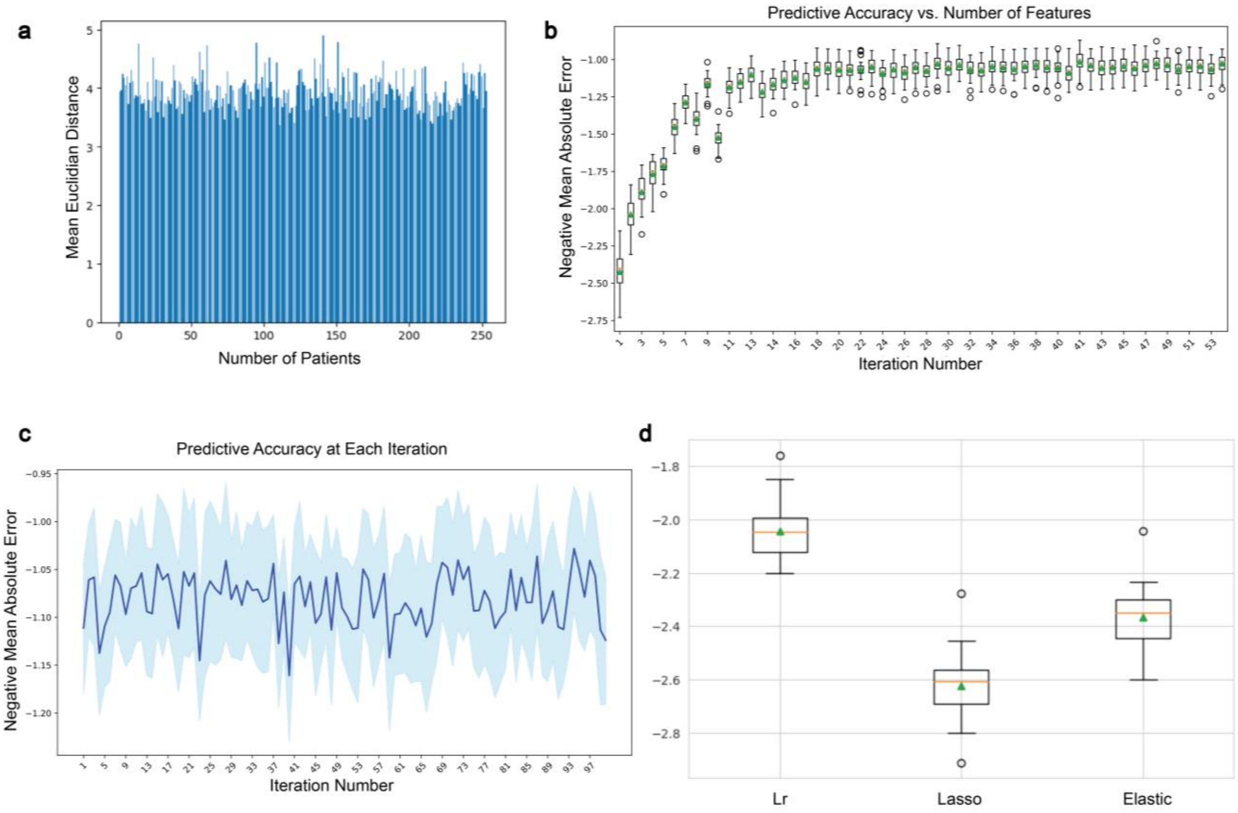

Figure S2. Details of similarity analysis and feature selection using decision model, related to Fig. 1-4 **a)** Mean pairwise distance in high dimensional feature space between patients for the day of admission. **b)** Predictive accuracy of the estimator with increasing number of features. For Scenario A, y axis denotes the -MAE of predicting the patient's SOFA score given the feature subset in repeated k-fold analysis. The predictive accuracy of the feature subset yielded such plateaus in all feature selection scenarios A-D, indicating that adding more features do not contribute to the model accuracy after a threshold, due to the limitations dictated by the sparsity of the dataspace. What differed between scenarios A-D was the negative MAEs, and the number of features it yields for its maximum accuracy. Overall, scenario A yielded the highest accuracy at lower number of features. **c)** Variations in repeated k-fold analysis for feature selection with GA (number_of_feature = 23). Each point shows the -MAE of the last generation of each 100 runs. This analysis further enables us to investigate the frequency of features being selected during the stochastic evolutionary process. **d)** Impact of using implicit feature selector as an estimator in GA wrapper. y axis denotes the -MAE in SOFA score predictions. lr: linear regression.

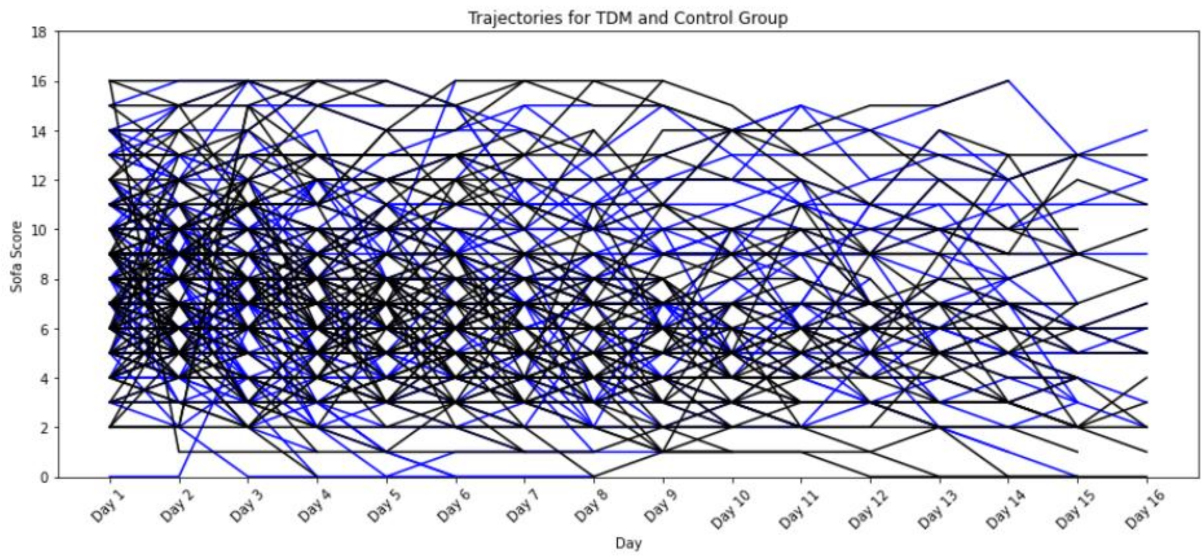

Figure S3. Fluctuations in patient SOFA score trajectories, related to Fig. 3. Patient health state jumps significantly between SOFA scores on a daily basis. Each curve represents a patient. Blue: TDM, Black: control.

Table S1. Detected pathogens data including type of pathogen and their occurrence in control and intervention groups responsible for sepsis episodes. Related to Fig. 4c-d. We fed the pathogen data to the decision model and found that pathogen-related data didn't make the cut very often, less than 10% of the time. But for the final feature set, we took the ones that were picked more than 10% of the time and threw them into another round of GA to make sure we covered any potential connections between them. Table is adapted from the original clinical study[2].

	**All patients[a] (n=253)**	**TDM (n=126)**	**No-TDM (n=127)**
**Gram-positive pathogen, No (%)**			
Staphylococcus aureus, Methicillin-susceptible	34 (6.7)	17 (6.2)	17 (7.4)
Staphylococcus aureus, Methicillin-resistant	1 (0.2)	1 (0.4)	0
Coagulase-negative staphylococci Methicillin-susceptible	26 (5.1)	14 (5.1)	12 (5.2)
Coagulase-negative staphylococci Methicillin-resistant	6 (1.2)	5 (1.8)	1 (0.4)
Streptococcus pneumoniae	3 (0.6)	1 (0.4)	2 (0.9)
Enterococcus faecalis	31 (6.1)	14 (5.1)	17 (7.4)
Enterococcus faecium	32 (6.3)	18 (6.5)	14 (6.1)
Other *Streptococcus* species	33 (6.6)	22 (8.0)	11 (4.8)
Other[b]	18 (3.6)	11 (4.0)	7 (3.0)
**Gram-negative pathogen, No (%)**			
Escherichia coli	86 (17.0)	50 (18.2)	36 (15.7)
Klebsiella species	59 (11.7)	30 (10.9)	29 (12.6)
Proteus species	25 (5.0)	14 (5.1)	11 (4.8)
Enterobacter species	27 (5.3)	14 (5.1)	13 (5.7)
Pseudomonas species	31 (6.1)	18 (6.5)	13 (5.7)
Serratia species	8 (1.6)	4 (1.5)	4 (1.7)
Citrobacter species	11 (2.2)	8 (2.9)	3 (1.3)
Acinetobacter species	2 (0.4)	1 (0.4)	1 (0.4)
Haemophilus species	3 (0.6)	1 (0.4)	2 (0.9)
Stenotrophomonas maltophilia	12 (2.4)	3 (1.1)	9 (3.9)
Other[c]	26 (5.1)	16 (5.8)	10 (4.3)
**Fungi, No (%)**			
Candida albicans	13 (2.6)	7 (2.5)	6 (2.6)
Other *Candida* species	8 (1.6)	2 (0.7)	6 (2.6)
Aspergillus species	3 (0.6)	2 (0.7)	1 (0.4)
Other[d]	6 (1.2)	1 (0.4)	5 (2.2)
**Other[e], No (%)**	1 (0.4)	1 (0.4)	0

[a] Multiple responses per patient possible

[b] Other includes other gram-positive bacteria (e.g., *Bacillus* species, *Corynebacterium* species, *Listeria species, Nocardia species*).

[c] Other includes other gram-negative bacteria (e.g., *Legionella*, *Moraxella*, *Neisseria*, *Salmonella*)

[d] Other includes *Phycomyces* species, *Coccidioides*, *Zygomyces*, *Rhizopus*, *Mucor*, and *Microsporum* species.

[e] Other includes *Mycobacteria species, Chlamydia species, Mycoplasma species*
